# Blockade of the CCL2–CCR2 axis attenuates fibrosis and parasite load in vesicular echinococcosis by inhibiting the PI3K-AKT pathway that regulates angiogenesis and hepatic stellate cell apoptosis

**DOI:** 10.1186/s13071-026-07306-3

**Published:** 2026-04-29

**Authors:** Bin Fan, Yuyu Ma, Menggen Meng, Jiahui Chen, Xinwei Qi, Yuqin Sun, Xuan Zhou, Haonan Wang, Xiumin Ma, Liang Wang

**Affiliations:** 1 Basic Medical College of Xinjiang Medical University, Ürümqi, Xinjiang PR China; 2 State Key Laboratory of Pathogenesis, Prevention and Treatment of High Incidence Diseases in Central Asia, Clinical Laboratory Center, Tumor Hospital Affiliated to Xinjiang Medical University, Ürümqi, 830011 Xinjiang PR China; 3 Medical Testing Center, The Fifth Affiliated Hospital of Xinjiang Medical University, Ürümqi, 830011 Xinjiang PR China; 4 Medical Testing Center, The First Affiliated Hospital of Xinjiang Medical University, Ürümqi, Xinjiang PR China

**Keywords:** Alveolar echinococcosis, CCL2–CCR2 signaling axis, PI3K-AKT signaling pathway

## Abstract

**Background:**

The C-C motif chemokine ligand 2 (CCL2)–C-C chemokine receptor type 2 (CCR2) signaling axis is critically involved in angiogenesis and cellular invasion; however, the therapeutic potential of its targeted blockade on alveolar echinococcosis (AE) remains largely unexplored. This study aimed to determine whether RS504393, a selective CCR2 antagonist, can inhibit the progression of AE by blocking the CCL2–CCR2 axis and modulating its downstream pathogenic mechanisms.

**Methods:**

In this study, bioinformatics analysis of the GSE124362 dataset was combined with molecular docking simulations. Liver tissues from patients with AE and from *Echinococcus multilocularis*-infected mice were examined through histopathological and immunohistochemical staining methods. Pathological alterations and parasitic load were assessed by Western blot and quantitative real-time PCR (RT-PCR). An in vitro co-culture model involving *Echinococcus multilocularis* protoscolex-stimulated endothelial progenitor cells (EPCs), JS1 hepatic stellate cells (JS1) and RAW264.7 macrophages was established to evaluate angiogenesis, fibrogenic activity and macrophage polarization.

**Results:**

Integrated bioinformatics and molecular docking analyses revealed CCL2–CCR2 overexpression and high-affinity binding during AE progression. Treatment with RS504393 significantly attenuated hepatic fibrosis, suppressed phosphoinositide 3-kinase (PI3K)-protein kinase B (AKT) pathway activation and reduced M2 macrophage polarization in central lesion tissue of *E. multilocularis*-infected mice . In vitro, RS504393 inhibited angiogenesis driven by EPCs, induced apoptosis in JS1 cells, and redirected macrophage polarization from the M2 towards a more anti-parasitic phenotype.

**Conclusions:**

CCR2 blockade with RS504393 attenuates AE progression by inhibiting PI3K-AKT signaling, suppressing angiogenesis and inducing stellate cell apoptosis, collectively reducing hepatic fibrosis and parasitic burden. Our findings provide a mechanistic rationale for repurposing CCR2 antagonists as a novel host-directed therapeutic strategy for echinococcosis.

**Graphical abstract:**

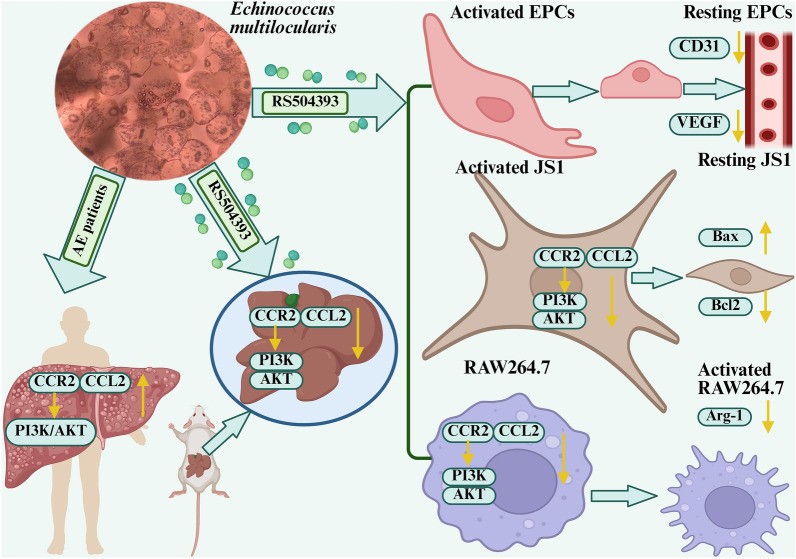

**Supplementary Information:**

The online version contains supplementary material available at 10.1186/s13071-026-07306-3.

## Background

China is a highly endemic region for echinococcosis, with alveolar echinococcosis (AE), caused by *Echinococcus multilocularis*, posing a serious public health threat and exhibiting a 10-year case fatality rate as high as 94%. The Xinjiang Uygur Autonomous Region of China is among the areas most severely affected.  *Echinococcus multilocularis* larvae primarily infest the liver, leading to tissue damage through direct invasion, cytotoxicity and mechanical compression. Pathologically, the larvae form cyst-like structures that exhibit cancer-like invasive growth, progressively destroying healthy hepatic tissue and often metastasizing to other organs. This aggressive behavior has earned AE the label of “a helminthic infection with malignant characteristics,” and it is ranked as one of the most lethal helminthic infections [1]. Early clinical symptoms are often subtle, resulting in delayed diagnosis until advanced stages when radical resection is no longer feasible. Even after partial hepatectomy, recurrence rates remain high [2, 3]. The progression of AE is further driven by immune cell infiltration, especially M2-polarized macrophages, which help create an immunosuppressive microenvironment that supports parasitic survival and accelerates hepatic fibrosis [4, 5]. Identifying novel therapeutic targets to disrupt this process is essential for improving outcomes in AE.

Chemokines are small secreted proteins that function through binding to G protein-coupled receptors (GPCRs) on target cells [6–9]. Among these, C-C motif chemokine ligand 2 (CCL2), also known as monocyte chemoattractant protein-1, is produced by a variety of cell types, including tumor cells, endothelial cells, fibroblasts and myeloid cells, and plays key roles in recruiting monocytes, T cells and natural killer (NK) cells to sites of inflammation and disease [10–12]. Its receptor, C-C chemokine receptor type 2 (CCR2), is a GPCR with high affinity for CCL2 [13, 14]. The CCL2–CCR2 axis is involved in pathological angiogenesis, tumor cell survival and immune cell recruitment across multiple diseases [15]. Upon ligand binding, CCR2 activates downstream signaling pathways such as the phospholipase C–protein kinase C (PLC-PKC) and phosphoinositide 3 kinase–protein kinase B (PI3K-AKT) pathways, which contribute to cell migration, survival and inhibition of apoptosis [16–20]. The CCL2–CCR2 axis also plays a central role in fibrotic diseases affecting the liver, pancreas and intestines, primarily by recruiting monocytes and macrophages that amplify inflammation and promote tissue remodeling [21]. In viral hepatitis, CCR2 helps establish an immunosuppressive microenvironment that accelerates fibrosis and supports viral persistence. During liver fibrosis, CCL2 acts synergistically with transforming growth factor-beta (TGF-β) to activate hepatic stellate cells (HSCs) and stimulate extracellular matrix deposition [22].

Although the CCL2–CCR2 pathway is well-studied in cancer and fibrotic diseases, its role in AE progression and the therapeutic potential of its blockade remain poorly understood. We hypothesized that inhibiting the CCL2–CCR2 axis with the selective antagonist RS504393 could mitigate fibrosis and angiogenesis in AE. Therefore, this study aimed to investigate the mechanistic contributions of CCL2–CCR2 signaling to AE pathogenesis and evaluate the therapeutic efficacy of RS504393 using both in vivo and in vitro models.

## Methods

### Bioinformatics analysis

Bioinformatic analysis was conducted using the GSE124362 dataset from the Gene Expression Omnibus (GEO) database (https://www.ncbi.nlm.nih.gov/geo/), which consists of liver tissues from six patients with AE, categorized into lesion and matched distal self-control groups. Differential gene expression analysis was carried out using R (v3.6.3; R Foundation for Statistical Computing, Vienna, Austria), applying thresholds of |log2FC|> 2 and an adjusted *P* value < 0.05 (Benjamini–Hochberg correction). Visualizations were generated with the ggplot2 package in R, and pathway enrichment analysis was subsequently performed using the clusterProfiler package.

### CCL2–CCR2 molecular docking and binding affinity assay

For molecular docking, the extracellular domain of CCR2 (PDB ID:1DOK, 2.5 Å resolution) and the CCR2 AlphaFold-predicted model (UniProt ID: P41597, AF-P41597-F1) were retrieved from the Protein Data Bank (PDB) archive and AlphaFold database, respectively. Structural preprocessing in AutoDock Tools 1.5.7 included water removal, hydrogen addition (pH 7.4, Gasteiger charges) and rigid fixation of CCR2 transmembrane helices (TM1–TM7) and extracellular loop 2 (ECL2), while allowing flexibility in CCL2’s N-terminal signal peptide (residues 1–10) and 40 s loop (residues 40–45) with five rotatable bonds. The docking grid was centered on CCR2’s ligand-binding domain (N-terminal extracellular region and ECL2; coordinates: *X* = 32.5 Å, *Y* = 22.1 Å, *Z* = 18.7 Å) to encompass the entire binding interface. AutoDock Vina was employed for protein–protein docking simulations, with minimum binding energy calculations used to identify optimal binding conformations.

### Human subjects

After obtaining written informed consent, paired tissue specimens were collected from 21 patients with AE (12 females/9 males) undergoing liver surgery, with a mean (± standard deviation [SD]) age of 45 ± 12 years. The AE diagnosis was confirmed in all patients by histopathological examination of liver tissues. For each patient, samples were obtained from both the central lesion tissue (CLT; < 0.5 cm from the lesion margin) and the distant lesion tissue (DLT; > 0.5 cm from the lesion margin). The study protocol was approved by the Ethics Committee of the First Affiliated Hospital of Xinjiang Medical University (Approval No.: 20190225–29; Urumqi, China).

### Animals

 Specific pathogen-free (SPF)-grade female BALB/c mice (6-8 weeks old) were provided by the First Affiliated Hospital of Xinjiang Medical University Animal Experiment Center. All experimental procedures were conducted under strict guidelines and the approval of the Institutional Animal Use and Care Committee and the Ethical Committee of the First Affiliated Hospital of Xinjiang Medical University (Approval No.: 20190225–29; Urumqi, China).

### *Echinococcus multilocularis* infection mouse model and specimen collection

The protocol for establishing the *E. multilocularis* infection model followed the method reported in an earlier publication [5]. Briefly, protoscoleces (PSCs) were aseptically isolated from Mongolian gerbils (*Meriones unguiculatus*) infected with *E. multilocularis* The viability of the PSCs was assessed using the 0.2% Trypan blue exclusion assay: 10 µl of PSC suspension was mixed with an equal volume of 0.2% Trypan blue solution, and the mixture incubated for 3 min and then examined under a light microscope. Unstained PSCs were considered to be viable, whereas blue-stained ones were deemed non-viable. Only PSC suspensions with viability exceeding 90% were used for animal infection.

Four experimental groups (*n* = 5 mice per group) were established: (i) a control group; (ii) *E. multilocularis* infection group (Em group); (iii) *E. multilocularis* infection + RS504393 intervention group (Em + RS504393 group); and (iv) control + RS504393 group (Control + RS504393 group).

 Both the *E. multilocularis* infection model group and the RS504393 intervention group (RS504393: a selective CCR2 antagonist) were inoculated intrahepatically with 2000 viable PSCs (> 90% viability) to establish a quantitative infection model. The PSCs for the infection model group were suspended in saline. Mice in the control group received an equivalent volume of sterile saline. Treatment in the RS504393 intervention group began 60 days post-infection. In this group, mice received daily intraperitoneal injections of RS504393 (2 mg/kg body weight) for 30 consecutive days and were euthanized 90 days after the completion of treatment (i.e. 150 days post-infection).

Samples of whole liver tissue and serum were taken from all mice. The five mice in the *E. multilocularis* infection model group were sacrificed at 8, 30, 60 and 90 days, post-infection, respectively to assess the successful establishment of the model. Five mice from each of the RS504393-treated groups were sacrificed at 150 days post-infection with *E. multilocularis*. At these same time points, five mice in the control group were sacrificed 1 day after being injected with normal saline.

### *Echinococcus multilocularis* PSC antigen preparation

The preparation of *E. multilocularis* PSC antigen (EmP) followed a previously reported method [5]. Briefly, PSCs were homogenized in liquid nitrogen, sonicated, rotated overnight at 4 °C and centrifuged. The supernatant was filter-sterilized and stored at − 80 °C until use.

### In vitro assessment of the effects of RS504393 on the viability and oxidative stress of PSCs

Protoscoleces were isolated from infected gerbil models and washed with Hanks' Balanced Salt Solution; morphologically intact, viable individuals were selected for the experiments. PSCs were seeded at a density of 100 per well in phenol red-free RPMI 1640 medium without fetal bovine serum and cultured at 24–30 °C. Three treatment groups were established: an RS504393-treated group (supplemented with 20 µg/ml RS504393), a positive control group (supplemented with 20 µg/ml albendazole), and a vehicle control group (cultured in medium containing 0.2% DMSO). The in vitro concentration of RS504393 was derived from its in vivo administration dose (2 mg/kg) based on standard pharmacokinetic parameters and the culture system volume, aiming to simulate the in vivo exposure levels. On days 1, 3, 5 and 7 of culture, the morphology and structure of the PSCs were observed under an optical microscope, and viability was assessed using the Trypan blue staining assay. On day 7 of culture, PSCs from each group were collected and stained with the reactive oxygen species (ROS) fluorescent probe DCFH-DA (final concentration: 2 µmol/l). This probe is hydrolyzed by intracellular esterases and then oxidized by ROS to generate the fluorescent compound DCF. The fluorescence intensity across groups was subsequently compared using fluorescence microscopy to indirectly reflect the level of oxidative stress within the PSCs.

### Detection by enzyme-linked immunosorbent assay

Serum levels of liver function markers—aspartate aminotransferase (AST), alanine aminotransferase (ALT), albumin (ALB), and total protein (TP)—and fibrosis biomarkers—hyaluronic acid (HA), laminin (LN), type IV Collagen (C-IV), and procollagen III peptide (PCIII)—were quantified using commercial enzyme-linked immunosorbent assay (ELISA) kits, strictly following the manufacturer's protocols (Additional file 6: Tables S1, S2).

### Cell culture

Primary mouse bone marrow-derived endothelial progenitor cells (BM-EPCs), the JS1 hepatic stellate cell line and RAW 264.7 macrophages were cultured in their respective complete growth media under standard conditions (37 °C, 5% CO_2_), as specified by the suppliers (Additional file 6: Table S1).

### CCK-8 cytotoxicity screening

The BM-EPCs, JS1, and RAW 264.7 cells were seeded in 96-well plates at optimized densities (1 × 10^5^ cells/well for all cell types) and allowed to adhere for 24 h. Cells were then treated with an EmP concentration gradients (30, 50, 100, 150 µg/ml for BM-EPCs/JS1/RAW 264.7) or RS504393 gradient (5, 10, 20, 30 µmol/l for BM- EPCs/JS1/RAW 264.7) in triplicate. Following a 48-h incubation, 10 µl CCK-8 reagent was added to each well and the well incubated for 2 h at 37 °C. Absorbance was quantified at 450 nm using a microplate reader. Optimal concentrations were determined by analyzing relative cell viability (%), resulting in final working concentrations of 80 µg/ml EmP for BM-EPCs/JS1, 100 µg/ml EmP for RAW 264.7, 10 µmol/l RS504393 for BM-EPCs/RAW 264.7 and 5 µmol/l RS504393 for JS1 (Additional file 6: Table S1).

### Annexin V-fluorescein isothiocyanate apoptosis detection assay

Apoptotic rates were quantified using the FITC Annexin V Apoptosis Detection Kit I (BD Biosciences, San Jose, CA, USA). JS1 cells were seeded in 6-well plates (1 × 10^6^ cells/well) and allowed to adhere for 24 h prior to RS504393 treatment. Following a 24-h drug exposure, cells were washed twice with ice-cold phosphate buffered saline (PBS), resuspended in 1 × Binding Buffer (1 × 10^6^ cells/ml) and aliquoted (1 × 10^5^ cells/tube) for staining. The cells were then incubated with 5 µl fluorescein isothiocyanate (FITC) Annexin V and 5 µl propidium iodide (PI) in darkness (15 min, room temperature), followed by the addition of 400 µl Binding Buffer. Samples were analyzed within 1 h using the FACSCanto II flow cytometer (BD Biosciences), with the apoptotic populations defined as follows: FITC Annexin V^+^/PI^−^ (early apoptosis), FITC Annexin V^+^/PI^+^ (late apoptosis/necrosis) and FITC Annexin V^−^/PI^−^ (viable/non-apoptotic) (Additional file 6: Table S1).

### Histopathological analysis

The liver tissues obtained from patients with AE and from the *E. multilocularis*-infected mice model in each experimental group were initially subjected to a dehydration process along an alcohol gradient, then subsequently embedded in paraffin. Sections with a thickness of 5 µm were meticulously prepared from the embedded tissues and then stained using hematoxylin–eosin (H&E) and the Sirius red staining kit, respectively, following the standard staining procedures (Additional file 6: Tables S1, S3).

### Immunohistochemistry analysis

For immunohistochemistry (IHC), 5-µm paraffin-embedded tissue sections were deparaffinized, rehydrated, and subjected to antigen retrieval before incubation with primary antibodies. Horseradish peroxidase conjugated secondary antibodies were applied, followed by 3,3′-diaminobenzidine chromogenic development (100 µL DAB working solution/section) to visualize antigen–antibody complexes. Detailed protocols for primary antibody application are comprehensively documented in Additional file 6: Table S3 to ensure experimental reproducibility. (Additional file 6: Tables S1, S3).

### RNA isolation and real-time PCR

Total RNA was isolated from specimens obtained from the patients with AE, *E. multilocularis*-infected mouse models and EmP-stimulated in vitro cell cultures, using TRIzol® Reagent (Invitrogen, Thermo Fisher Scientific, Waltham, MA, USA). RNA integrity was verified by agarose gel electrophoresis before reverse transcription with the PrimeScript™ RT Reagent Kit with gDNA Eraser (Takara Bio., Kusatsu, Shiga, Japan). The PrimeScript RT Master Mix (Perfect Real Time) Kit (Takara Bio.) was used for the reverse transcription reaction. Each reaction uses 1000 ng total RNA as a template, and the reaction volume is 10 µl. Quantitative real-time PCR (qRT-PCR) amplification was performed using TB Green® Premix Ex Taq™ II on an Applied Biosystems 7500 Fast Real-Time PCR System (Applied Biosystems, Thermo Fisher Scientific), with glyceraldehyde-3-phosphate dehydrogenase (GAPDH) messenger RNA (mRNA) expression serving as the endogenous normalization control. Gene-specific primer pairs (Additional file 6: Table S4) were designed using Primer-BLAST. The relative mRNA expression level of the gene was calculated using the 2^^−ΔΔCt^ method and standardized by the reference gene GAPDH Primer details (Additional file 6: Tables S1, S4).

### Western blot analysis

Total protein lysates were extracted from the AE clinical specimens, *E. multilocularis*-infected mouse model cohorts and EmP-stimulated in vitro cell cultures using RIPA lysis buffer supplemented with protease/phosphatase inhibitors. Protein concentrations were quantified via the BCA assay prior to separation in 10–12% gradient sodium diodecyl sulfate-polyacrylamide gel electrophoresis (SDS-PAGE) gels and electrophoretic transfer to PVDF membranes (loading amount: 10 µl; protein content: 20 µg). Membranes were blocked with 5% non-fat dried milk in TBST wash buffer (25 °C, 1.5 h), then incubated with primary antibodies at 4° C for 16 h (dilutions are shown in Additional file 6: Table S5), followed by incubation with horseradish peroxidase (HRP)-conjugated secondary antibodies at 25 °C for 2 h. Chemiluminescent signals were developed using SuperSignal™ West Pico PLUS ECL Substrate and quantified via densitometric analysis in Image Lab™ Software (v6.1; Bio-Rad Laboratories, Hercules, CA, USA), with GAPDH serving as the loading control. Detailed protocols for primary antibody application are comprehensively documented in Additional file 6: Table S5 to ensure experimental reproducibility (Additional file 6: Tables S1, S5).

### Statistical analysis

All experiments were performed with at least three independent biological replicates. Data were analyzed using SPSS Statistics version 29 (SPSS IBM Corp., Armonk, NY, USA) and GraphPad Prism® 9.0 software (GraphPad Software, San Diego, CA, USA). First, normality (Shapiro-Wilk test) and homogeneity of variance (Levene’s test) were assessed for all quantitative datasets. For data meeting the assumptions of normality and homogeneity of variance, comparisons between two groups were performed using two-tailed Student’s t-tests, while comparisons among three or more groups were analyzed by one-way analysis of variance (ANOVA) followed by Tukey's post hoc test. If the data failed to meet the assumptions for parametric tests, appropriate non-parametric tests were applied: the Mann–Whitney U-test for comparisons between two groups, and the Kruskal–Wallis test for comparisons among three or more groups. Statistical significance was defined as *P* < 0.05 (*), *P* < 0.01 (**) and *P* < 0.001 (***). Exact *P*-values are provided for critical comparisons in the figure legends.

## Results

### Integrated bioinformatics and structural analyses delineate CCL2–CCR2 interaction with high binding affinity

**Fig. 1:**
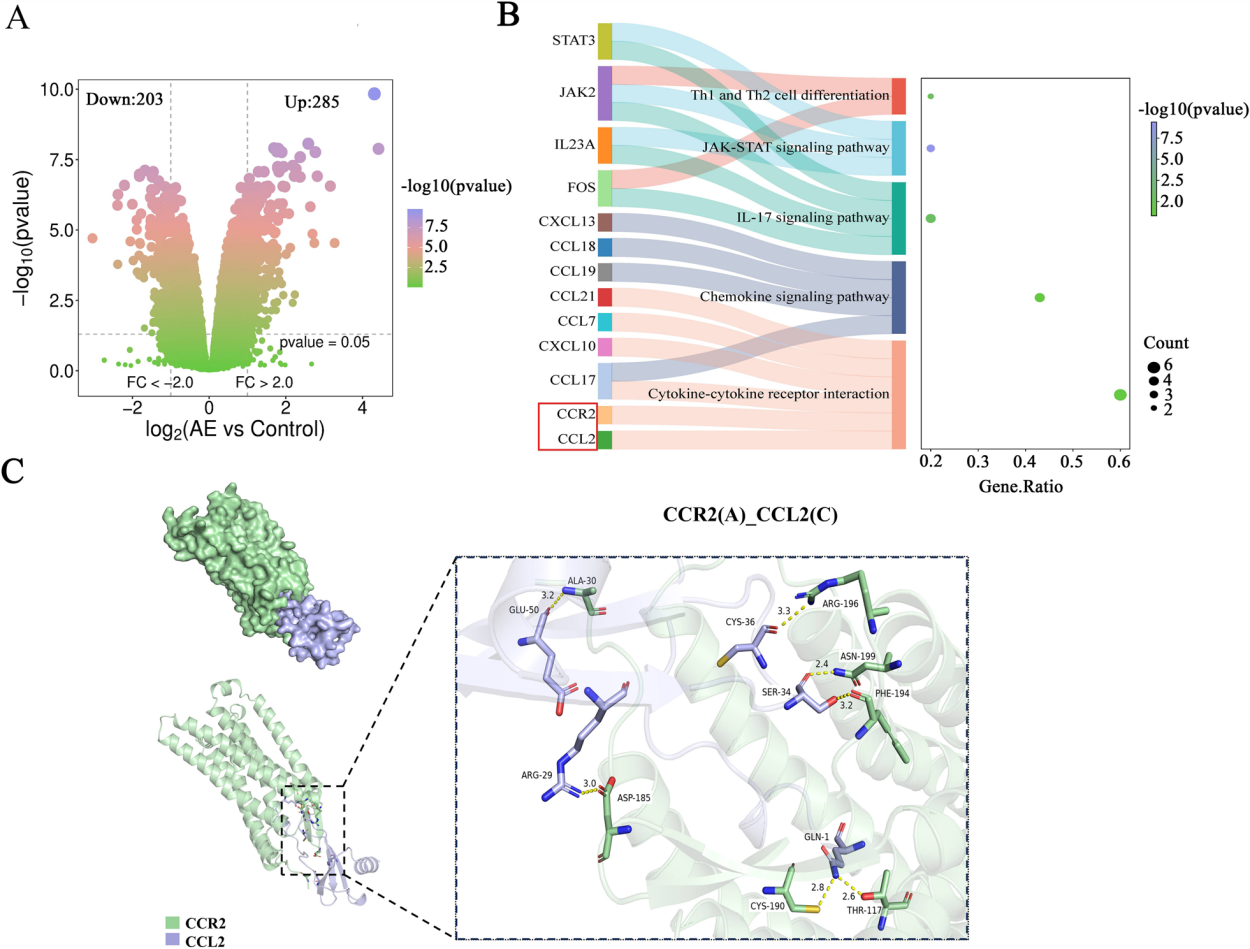
Bioinformatic analysis reveals high-affinity CCL2–CCR2 interaction. **A** Volcano plot of differentially expressed genes in the GSE124362 dataset. **B** KEGG (Kyoto Encyclopedia of Genes and Genomes) pathway enrichment analysis of the GSE124362 dataset, highlighting chemokine–chemokine receptor interaction as a top enriched pathway. **C** Molecular docking model of CCL2–CCR2 interaction, illustrating hydrogen-bond networks between critical residues (CCR2: GLU50, ARG29, CYS36; CCR2: ASP185, ARG196, ASN199). AE, Alveolar echinococcosis; CCL2, C-C motif chemokine ligand 2; CCR2, C-C chemokine receptor type 2; FC, fold-change

Transcriptomic profiling of the GSE124362 dataset identified 285 significantly upregulated and 203 downregulated genes under stringent thresholds (LogFC|> 2 and adjusted *P* < 0.05), as visualized by volcano plot (Fig. 1a). The KEGG (Kyoto Encyclopedia of Genes and Genomes) pathway enrichment analysis revealed predominant clustering of differentially expressed genes (DEGs) in chemokine signaling pathways, particularly highlighting the chemokine–chemokine receptor interaction axis (Fig. 1b). Further analysis specifically within this pathway showed that CCR2 was the top-ranked upregulated DEG (log2FC = 3.5, adjusted *P* = 0.0012). Concurrently, its specific receptor CCR2 was also among the most significantly upregulated genes (ranked second, log2FC = 2.6). Given the well-established central role of the CCL2–CCR2 axis in mediating monocyte/macrophage recruitment and driving hepatic fibrosis, and its identification as the most prominently dysregulated ligand–receptor pair in our pathway analysis, we therefore selected it as the key target for subsequent in-depth functional and mechanistic investigation. Molecular docking simulations demonstrated high-affinity binding between CCL2 and CCR2 mediated by an extensive hydrogen-bond network: critical residues in CCL2 (GLU50, ARG29, CYS36, SER34, GLN1) formed hydrogen bonds with CCR2 residues (ALA30, ASP185, ARG196, ASN199, PHE194, CYS190, THR117), establishing a stable interaction interface (Fig. 1c).

### Histopathological and molecular analyses revealed marked activation of the CCL2–CCR2 axis accompanied by PI3K-AKT signaling in hepatic AE lesions

**Fig. 2:**
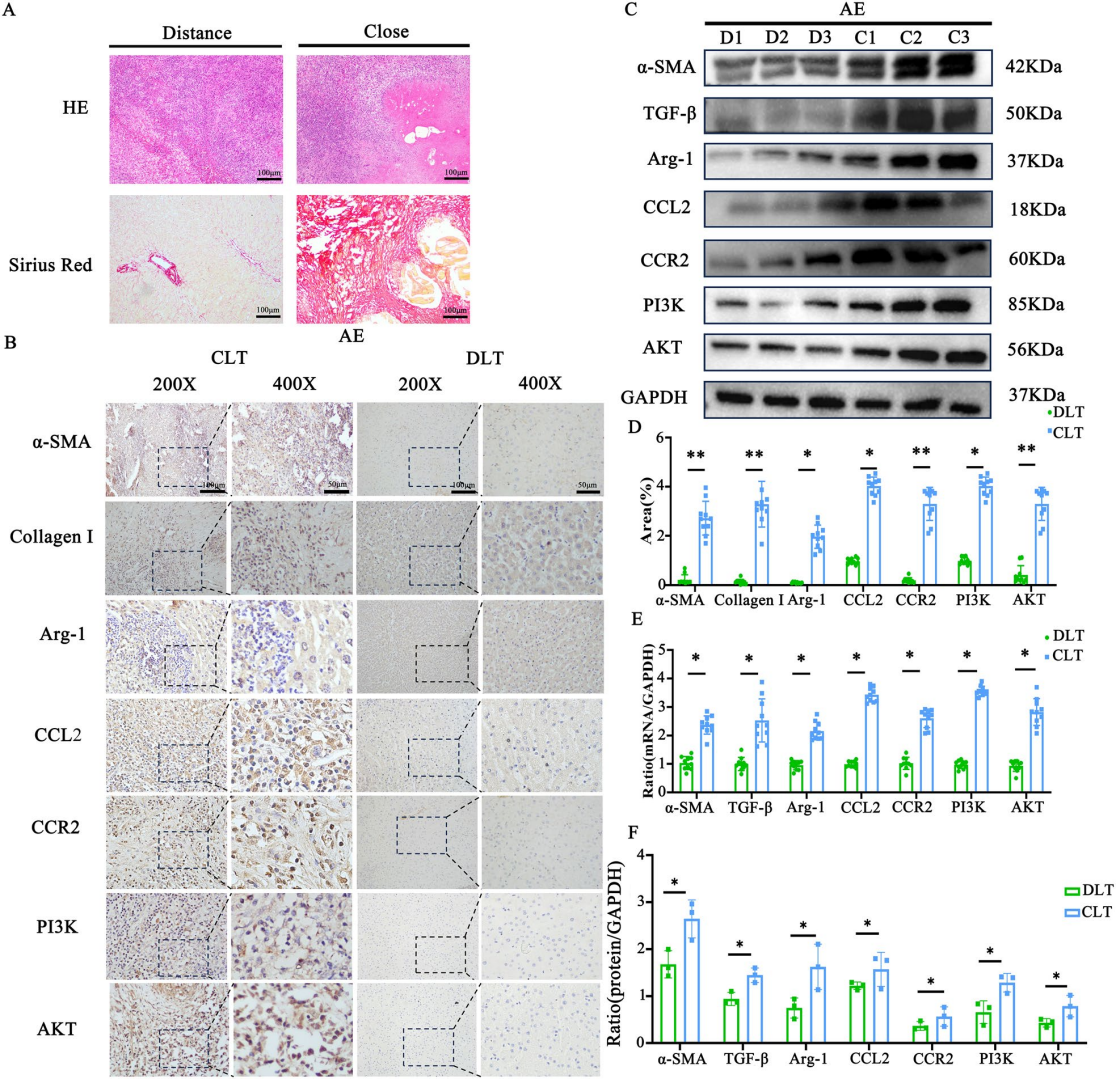
Histopathological and molecular profiling of hepatic lesions in patients with AE. **A** Representative HE and Sirius red staining of AE liver tissues, showing infiltrative cyst structures and collagen deposition (*n* = 10 per group). Scale bars: 200 µm. Original magnification ×200. **B** Immunohistochemistry (IHC) staining for fibrotic markers (α-SMA, Collagen I), M2 macrophage marker arginase-1 (Arg-1), and CCL2–CCR2/PI3K-AKT pathway components (*n* = 10 per group). Scale bars: 200 µm. Original magnification ×200. **C** Western blotting analysis of α-SMA, TGF-β, Arg-1, CCL2, CCR2, PI3K and AKT protein expression. **D**–**F** Quantitative analysis of results from IHC, quantitative real-time PCR and western blotting, demonstrating upregulated expression of fibrotic, angiogenic and signaling markers in CLT regions compared to DLT regions (*n* = 10 per group). Data are expressed as the mean ± standard deviation. Asterisks indicate statistically significant difference at *P < 0.05, **P < 0.01 and ***P < 0.001 by the two-tailed paired t-test. AE, Alveolar echinococcosis; AKT, Protein kinase B; Arg-1, arginase-1; α-SMA, alpha-smooth muscle actin; CCL2, C-C motif chemokine ligand 2; CCR2, C-C chemokine receptor type 2; CLT, central lesion tissue; DLT, distal lesion tissue; GAPDH, glyceraldehyde-3-phosphate dehydrogenase; HE, hematoxylin–eosin stain; mRNA, messenger RNA; PI3K, phosphoinositide 3-kinase; TGF-β, transforming growth factor-beta

Pathological examination of hepatic lesions from patients with AE revealed the characteristic disruption of lobular architecture, with multifocal parasitic vesicles and extensive fibrous capsules surrounded by dense macrophage infiltration (Fig. 2a). Sirius red staining further confirmed advanced collagen deposition within the CLT (Fig. 2a). Immunohistochemistry studies showed significant upregulation of alpha-smooth muscle actin (α-SMA), collagen I, arginase-1 (Arg-1), CCL2, CCR2, PI3K, AKT, matrix metalloproteinase (MMP)1 and MMP8 in the CLT compared to DLT (all *P* < 0.05; Fig. 2b, d; Additional file 1: Figure S1A–C). Consistent with the IHC findings, western blot analysis indicated that protein levels of these markers were markedly increased in the CLT (*P* < 0.05; Fig. 2c, f). Significantly higher levels of phosphorylated PI3K and p-AKT (p-PI3K and p-AKT) were found in CLT relative to the total proteins (*P* < 0.01; Additional file 2: Fig. S2A, B). Furthermore, qRT-PCR results confirmed elevated mRNA expression of all seven markers in the same regions (*P* < 0.05; Fig. 2e). These multi-method results consistently demonstrated concurrent activation of the CCL2–CCR2 axis and PI3K-AKT signaling within the fibrotic microenvironment of AE lesions, suggesting a potential mechanistic link in AE-associated hepatic fibrogenesis.

### RS504393 reduces hepatic parasitic load and fibrosis progression in mice infected with *E. multilocularis* by blocking the CCL2–CCR2 axis

**Fig. 3:**
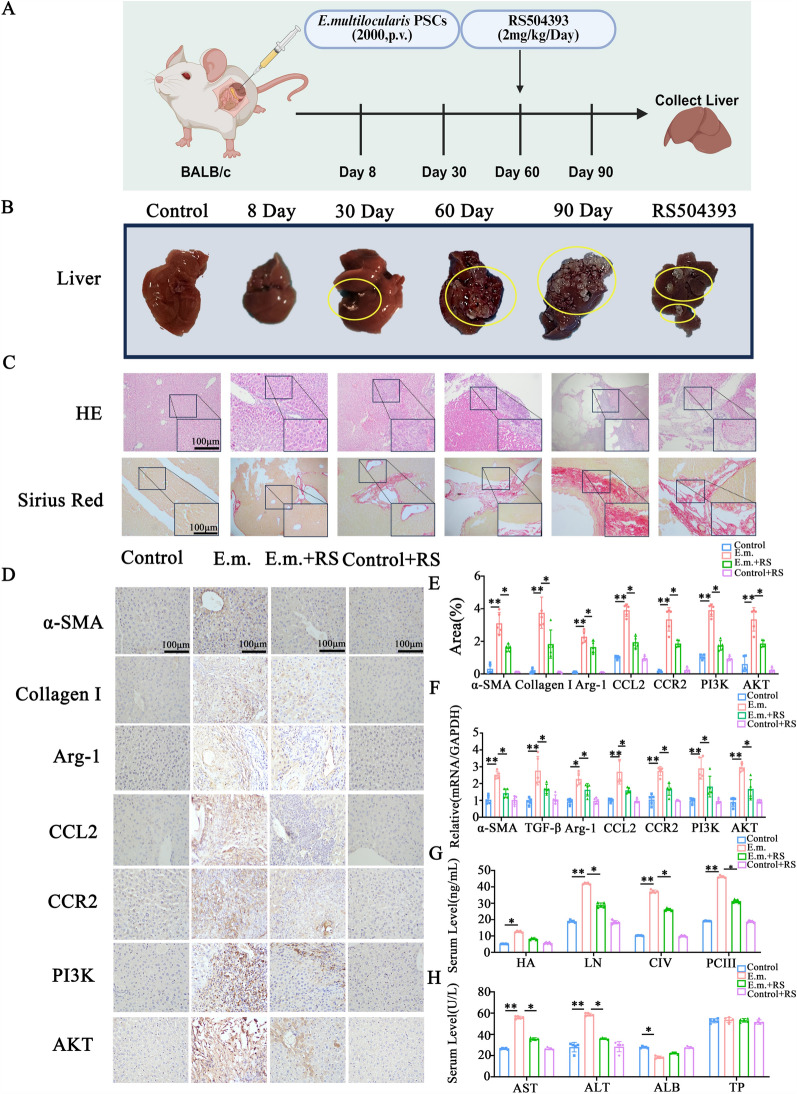
The selective CCR2 antagonist RS504393 attenuated hepatic fibrosis in *Echinococcus multilocularis*-infected mice. **A** Experimental time line of *E. multilocularis* infection and RS504393 intervention (intraperitoneal administration from day 60–90 post-infection). **B** Gross liver morphology of mice in control, *E. multilocularis*-infected (8-, 30-, 60- and 90-days post-infection) and RS504393-treated groups, showing reduced cyst burden and fibrotic lesions post-intervention. **C** Representative HE and Sirius red staining of liver tissues: H&E reveals attenuated parenchymal necrosis and inflammatory infiltration, while Sirius red demonstrates decreased collagen deposition (*n* = 5 per group). Scale bars: 200 µm. Original magnification ×200. **D** Immunohistochemistry staining for fibrotic markers (α-SMA, Collagen I), Arg-1 and CCL2–CCR2/PI3K-AKT pathway components across groups (*n* = 5 per group). Scale bars: 200 µm. Original magnification ×200. **E**, **F** Quantitative analysis of IHC and quantitative real-time PCR results, showing significant downregulation of α-SMA, Collagen I, Arg-1, CCR2, CCR2, PI3K and AKT in RS504393-treated mice (*n* = 5 per group). **G**, **H** Serum levels of hepatic fibrosis markers (HA, LN, PCIII, C-IV) and liver function indices (AST, ALT, ALB, TP) measured by enzyme-linked immunosorbent assay, with RS504393 treatment significantly reducing fibrosis progression and hepatocyte injury (*n* = 5 per group). Data are presented as the mean ± standard error of the mean. Statistical comparisons among groups were performed using one-way analysis of variance with appropriate post hoc tests for multiple comparisons. Asterisks indicate statistically significant difference at *P < 0.05, **P < 0.01 and ***P < 0.001. ALB, albumin; ALT, alanine aminotransferase; AST, aspartate aminotransferase; C-IV, type IV collagen; E.m., *Echinococcus multilocularis*; HA, hyaluronic acid; LN, laminin; PCIII, procollagen III peptide; PSCs, protoscoleces; RS, RS504393; TP, total protein; see caption to Fig. 2 for other abbreviations

Following the blockade of the CCL2–CCR2 signaling axis by the selective CCR2 antagonist RS504393, we observed a significant reduction in parasitic load and hepatic fibrosis in mice infected with *E. multilocularis*. Upon intraperitoneal administration of RS504393, initiated at 60 days post-infection for 30 consecutive days (Fig. 3a), the hepatic parasitic burden in infected mice was markedly reduced compared to the untreated infection group, evidenced by an approximate 50% decrease in cyst volume (Fig. 3b). H&E staining revealed that RS504393 treatment significantly attenuated inflammatory cell infiltration, reduced areas of hepatocellular necrosis, mitigated the disruption of normal lobular architecture and resulted in more orderly hepatocyte arrangement (Fig. 3c). Sirius red staining demonstrated a notable decrease in orange–red collagen deposition, indicating reduced hepatic fibrosis; this was characterized by thinner and sparser fibrous septa, shortened and narrowed fibrotic intervals, diminished pseudo lobule formation and improved architectural integrity and continuity of the liver tissue (Fig. 3c). Furthermore, following RS504393 intervention, IHC showed a reduction in the positive staining areas for α-SMA and Collagen I (*P* < 0.05), and qRT-PCR confirmed significant downregulation of α-SMA and Collagen I mRNA expression (*P* < 0.05; Fig. 3d–f). These findings suggest that inhibition of the CCL2–CCR2 axis effectively alleviated the pathological progression of hepatic fibrosis, improved liver histoarchitecture and enhanced functional status in *E. multilocularis*-infected mice. RS504393 treatment significantly reduced serum levels of the following hepatic fibrosis markers: HA (12.64 ± 0.46 vs. 5.16 ± 0.15 ng/ml, *P* < 0.05), LN (42.06 ± 0.42 vs. 19.20 0.73 ng/ml, *P* < 0.05), PCIII (46.20 ± 0.63 vs. 9.14 ± 0.16 ng/ml, *P* < 0.05) and C-IV (37.18 ± 0.80 vs. 10.26 ± 0.22 ng/ml, *P* < 0.05) (Fig. 3g). Concurrently, CCL2–CCR2 axis blockade effectively decreased the levels of AST (55.80 ± 1.30 vs. 35.60 ± 1.14 U/l; *P* < 0.05) and ALT (58.60 ± 1.36 vs. 36.00 ± 0.71U/l; *P* < 0.05), indicating a significant improvement in liver function (Fig. 3h). Additionally, our study found that RS504393 intervention markedly downregulated the PI3K-AKT signaling pathway, as evidenced by reduced IHC-positive areas for PI3K and AKT (*P* < 0.05) and significantly lower mRNA levels (*P* < 0.05; Fig. 3d–f).

### RS504393 inhibits angiogenesis activity in *E. multilocularis*-infected mice by blocking the CCL2–CCR2 axis

**Fig. 4:**
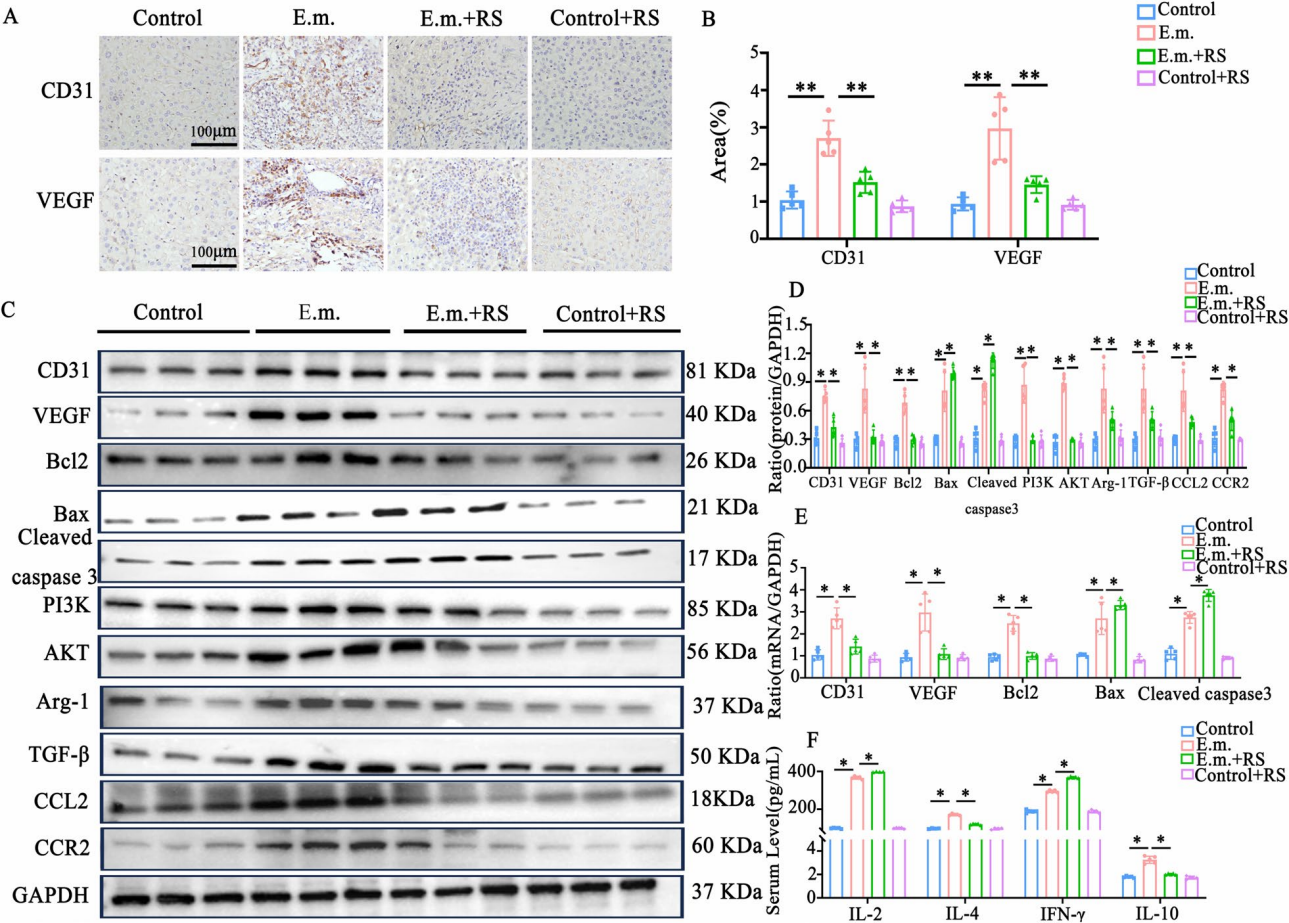
The selective CCR2 antagonist RS504393 suppressed angiogenesis and modulates multifactorial signaling in *Echinococcus multilocularis*-infected mice. **A** Immunohistochemistry (IHC) staining of CD31 and VEGF in liver tissues from control, *E. multilocularis*-infected and RS504393-treated groups, showing reduced vascular density post-treatment (*n* = 5 per group). Scale bars: 200 µm. Original magnification ×200. **B** Quantitative analysis of CD31/VEGF IHC staining intensity. **C** Western blotting (WB) analysis of angiogenesis markers (CD31, VEGF), apoptosis regulators (Bcl-2, Bax, Cleaved-caspase-3), fibrotic mediators (TGF-β, Arg-1) and CCL2–CCR2/PI3K-AKT pathway components (*n* = 5 per group). **D** Statistical results of WB quantitative analysis (*n* = 5 per group). **E** Quantitative real-time PCR validation of CD31, VEGF, Bax/Bcl-2 ratio,and cleaved caspase-3 mRNA levels (*n* = 5 per group). **F** Enzyme-linked immunosorbent assay quantification of serum cytokines (IL-2, IL-4, IFN-γ, IL-10), revealing RS504393-induced reversal of immunosuppressive Th2/IL-10 dominance (*n* = 5 per group). Data are presented as the mean ± standard error of the mean. Statistical comparisons among groups were performed using one-way analysis of variance with appropriate post hoc tests for multiple comparisons. Asterisks indicate statistically significant difference at *P < 0.05, **P < 0.01 and ***P < 0.001. Bax, Bcl-2-associated X protein; BCL-2, Bcl-2 B-cell lymphoma 2; CD31, platelet endothelial adhesion molecule; E.m., *Echinococcus multilocularis*; IFN, interferon; IL, interleukin; RS, RS504393; Th2, T helper 2 cells; VEGF, vascular endothelial growth factor; see caption to Fig. 2 for other abbreviations

RS504393 inhibited angiogenic activity in mice with *E. multilocularis* by blocking the CCL2–CCR2 axis. Pathological vasculature provides essential nutrients and waste exchange channels for the growth, maturation and invasion of *E. multilocularis* metacestodes. Therefore, we further investigated the effect of RS504393 on the expression levels of the angiogenesis-related factors CD31, a cell–cell adhesion and signaling protein, and vascular endothelial growth factor (VEGF), in the hepatic lesion tissues of infected mice. Following RS504393 intervention, a significant reduction was observed in the IHC-positive area for CD31 and VEGF (*P* < 0.01), along with decreased protein expression (*P* < 0.05) and markedly downregulated mRNA levels of CD31 and VEGF (*P* < 0.05; Fig. 4a–d). Furthermore, in the hepatic lesions of RS504393-treated mice, the expression of pro-apoptotic indicators Bax and Cleaved-caspase-3 was significantly increased at both protein and mRNA levels (*P* < 0.05), while the expression of the anti-apoptotic protein Bcl-2 was decreased (*P* < 0.05; Fig. 4d, e). The PI3K/AKT signaling pathway plays a critical role in regulating both apoptosis and angiogenesis. Western blot and qPCR analyses demonstrated that RS504393 treatment led to reduced protein and mRNA expression of PI3K and AKT (*P* < 0.05). RS504393 intervention significantly decreased the levels of p-PI3K and p-AKT relative to their total protein forms (*P* < 0.05; Additional file 2: Figure S2C, D). Concurrently, results showed that the protein and mRNA expression of Arg-1, a surface marker for M2 macrophages, was decreased following RS504393 intervention (*P* < 0.05). ELISA revealed that cytokine secretion in orbital blood of mice was altered after RS504393 treatment: levels of IL-4 (171.4 ± 2.70 vs. 120.0 ± 3.94; *P* < 0.05) and IL-10 (3.30 ± 0.25 vs. 2.03 ± 0.06; *P* < 0.05) were decreased, while levels of IL-2 (367.2 ± 5.76 vs. 398.8 ± 2.17; *P* < 0.05) and interferon-gamma (IFN-γ; 295.2 ± 5.93 vs. 368.6 ± 4.39; *P* < 0.05) were increased (Fig. 4f).

### RS504393 attenuates hepatic fibrosis via coordinated suppression of angiogenesis, induction of apoptosis and macrophage repolarization

**Fig. 5:**
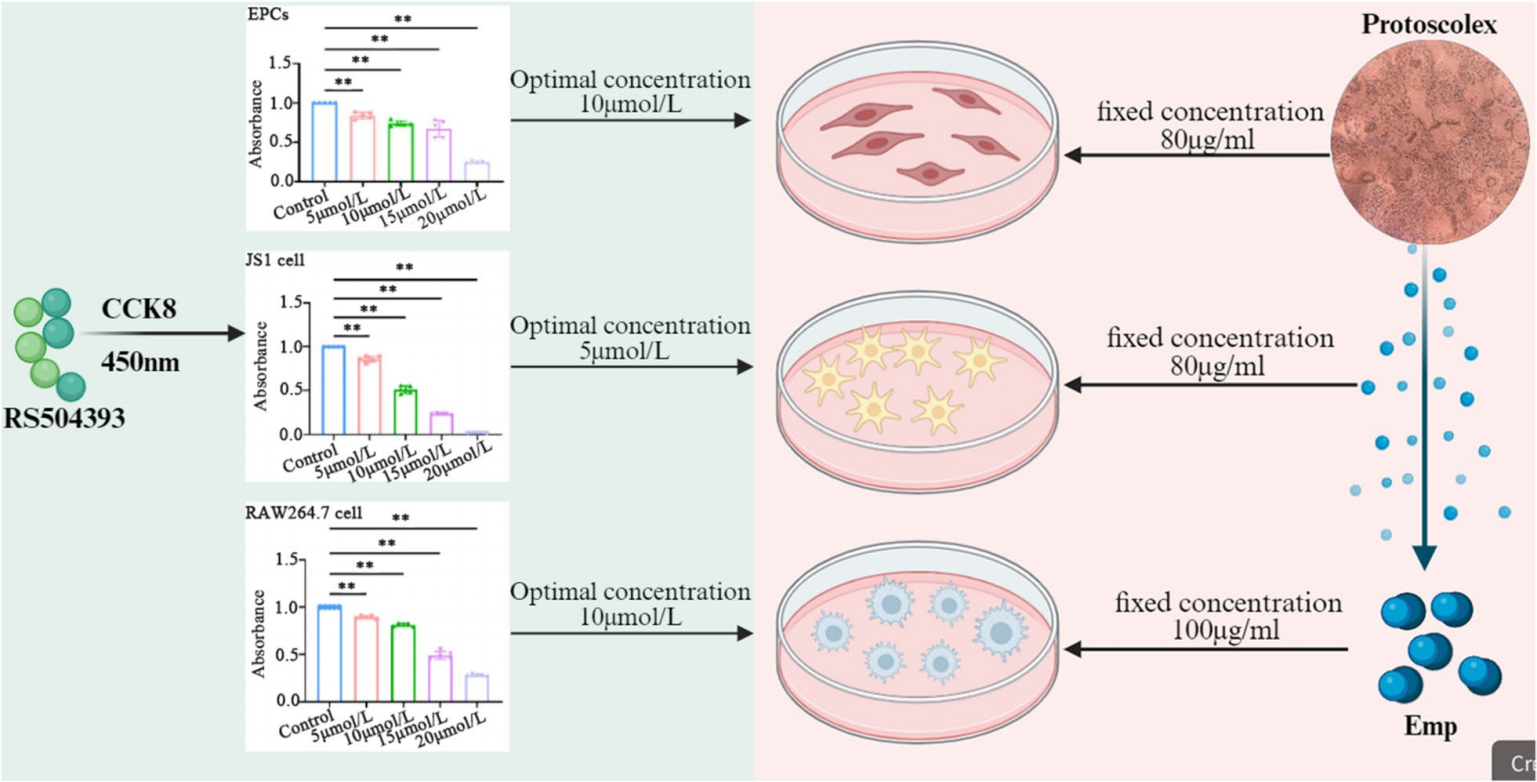
Schematic diagram of the in vitro co-culture system integrating EmP with EPCs, JS1, and RAW264.7 macrophages. To investigate the direct effects of EmP on key cell types within the pathological microenvironment, we established independent co-culture systems in which EPCs, JS1 and RAW264.7 were individually exposed to EmP. This schematic diagram summarizes the key experimental processes involved in the application of the CCL2–CCR2 axis inhibitor RS504393 in these systems. Asterisks indicate statistically significant difference at *P < 0.05, **P < 0.01. CCK-8, Cell Counting Kit-8; CCL2–CCR2, C-C chemokine receptor type 2–C-C chemokine receptor type 2 axis; EmP, *E. multilocularis* PSC antigen; EPCs, human umbilical vein endothelial progenitor cells; JS1, murine hepatic stellate cells; RAW264.7, murine macrophage cells

**Fig. 6:**
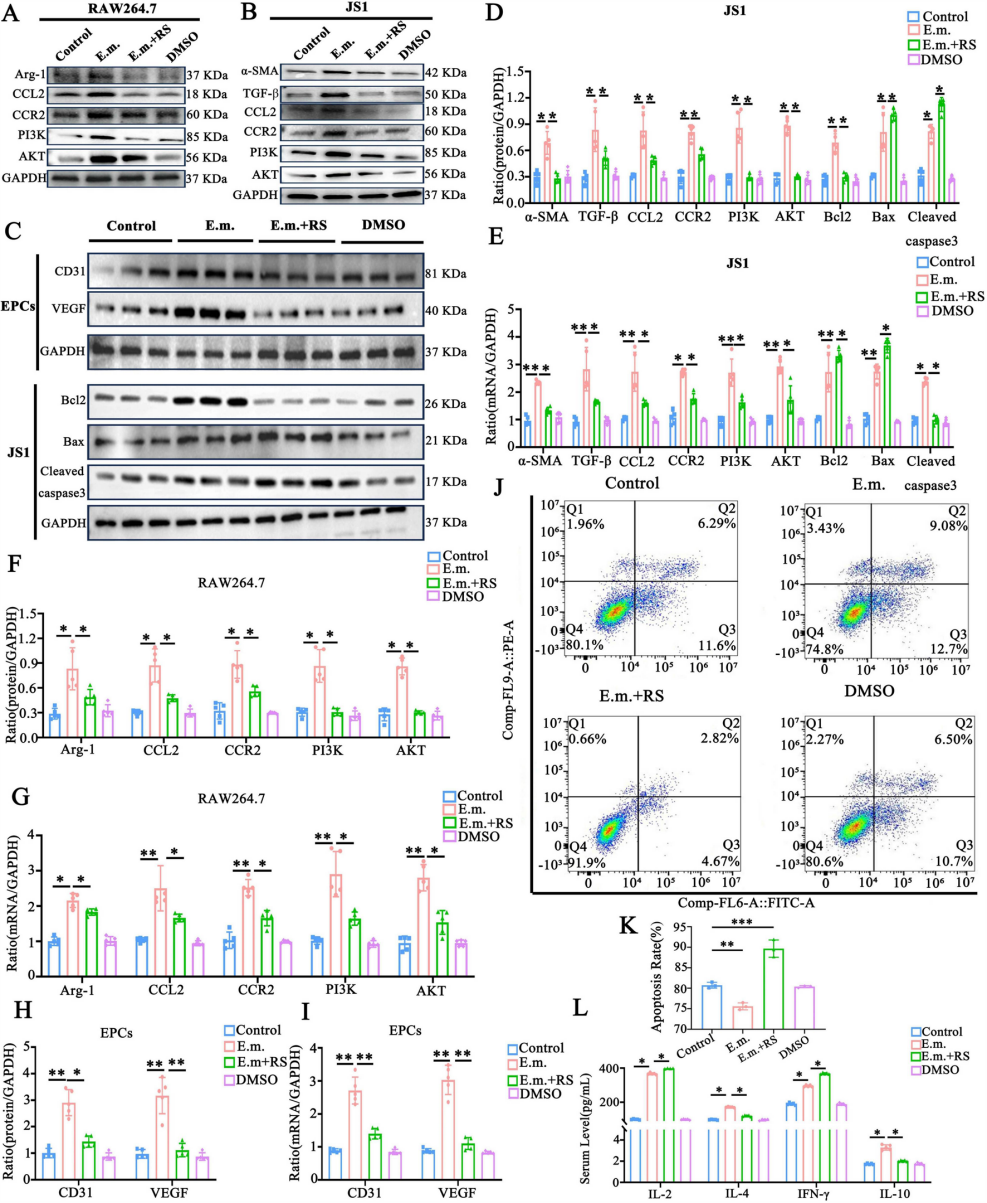
The selective CCR2 antagonist RS504393 attenuated hepatic fibrosis via coordinated suppression of angiogenesis, induction of apoptosis and macrophage repolarization. **A** Western blotting (WB) analysis of Arg-1, CCL2, CCR2, PI3K and AKT in RAW264.7 macrophages across control, *Echinococcus multilocularis*-infected and RS504393-treated groups (*n* = 5 per group). **B** WB of Arg-1, CCR2, CCR2, PI3K and AKT in JS1 hepatic stellate cells (*n* = 5 per group). **C** WB of angiogenesis markers (CD31, VEGF) in EPCs and apoptosis regulators (Bcl-2, Bax, cleaved caspase-3) in JS1 cells (*n* = 5 per group). **D**, **E** Quantitative analysis of WB and quantitative real-time PCR (qRT-PCR) results in JS1 cells, showing RS504393-mediated suppression of fibrotic markers and activation of apoptosis (*n* = 5 per group). **F**, **G** WB and qRT-PCR quantification in RAW264.7 macrophages, demonstrating reduced Arg-1 and CCL2–CCR2/PI3K-AKT pathway inhibition. **H**, **I**. WB and qRT-PCR analysis in EPCs, confirming attenuated angiogenesis (CD31, VEGF) (*n* = 5 per group). **J**, **K** Flow cytometric quantification of apoptosis in JS1 cells (Annexin V/PI staining) and statistical results (*n* = 3 per group). **L** Enzyme-linked immunosorbent assay of cytokines (IL-2, IL-4, IFN-γ, IL-10) in RAW264.7 supernatant, revealing RS504393-induced reversal of immunosuppressive IL-10 dominance (*n* = 5 per group). Data are presented as the mean ± standard error of the mean. Statistical comparisons among groups were performed using one-way analysis of variance with appropriate post hoc tests for multiple comparisons. Asterisks indicate statistically significant difference at *P < 0.05, **P < 0.01 and ***P < 0.001. See captions to Figs. 2–5 for abbreviations

To dissect the direct effects of EmP on key cellular players in the pathological microenvironment, we established independent co-culture models in which EPCs, JS1 or RAW264.7 cells were individually exposed to EmP. RS504393 intervention experiments in these parallel systems (Fig. 5) demonstrated that this CCL2–CCR2 axis inhibitor significantly modulated the responses of these distinct cell types. CCK-8 assays determined the optimal treatment concentrations of RS504393 to be 10 µmol/l for EPCs and RAW 264.7 cells, and 5 µmol/l for JS1 cells. In macrophages, Western blot and qRT-PCR analyses revealed that RS504393 significantly reduced the expression of the M2 polarization marker Arg-1 (*P* < 0.05), as well as of the protein and mRNA levels of the key PI3K-AKT signaling molecules, PI3K and AKT. RS504393 intervention significantly decreased the levels of p-PI3K and p-AKT relative to their total protein forms (*P* < 0.05; Fig. 6a, f, g; Additional file 2: Figure S2E–H), indicating its efficacy in suppressing the tendency of macrophages toward M2 polarization. In JS1, RS504393 treatment downregulated the protein and mRNA expression of fibrotic markers α-SMA and TGF-β (*P* < 0.05; Fig. 6b, d, e). Conversely, it induced a pro-apoptotic signature characterized by increased expression of Bax and Cleaved-caspase-3 and decreased expression of Bcl-2 at both the protein and mRNA levels (*P* < 0.05; Fig. 6c–e). Flow cytometry further confirmed a significant increase in the apoptosis rate of JS1 cells following RS504393 treatment (75.60 ± 0.87% vs. 89.67 ± 2.17%; *P* < 0.01) (Fig. 6j, k). ELISA assays showed that RS504393 treatment altered cytokine secretion in macrophage supernatants: levels of IL-4 (181.3 ± 1.70 vs. 115.0 ± 2.94 pg/ml; *P* < 0.05) and IL-10 (3.60 ± 0.15 vs. 2.01 ± 0.05 pg/ml; *P* < 0.05) were decreased, while levels of IL-2 (360.4 ± 3.70 vs. 380.6 ± 2.94 pg/ml; *P* < 0.05) and IFN-γ (305.2 ± 3.93 vs. 371.6 ± 2.39 pg/ml; *P* < 0.05) were increased (Fig. 6l). Regarding angiogenesis, the protein and mRNA expression of the key pro-angiogenic molecules CD31 and VEGF in EPCs was reduced following RS504393 intervention (*P* < 0.05; Fig. 6c, h, i). These systematic findings indicate that by blocking the CCL2–CCR2 axis, RS504393 not only inhibited PI3K-AKT pathway-mediated M2 polarization of macrophages but also mitigated the fibrotic phenotype and induced apoptosis in hepatic stellate cells, while coordinately suppressing the pro-angiogenic activity of EPCs. This multifaceted action ultimately underpins its multi-target mechanism against hepatic fibrosis.

## Discussion

Alveolar echinococcosis, caused by the larval stage of *E. multilocularis*, is a lethal zoonotic parasitic disease characterized by infiltrative hepatic growth of meta-cestodes. The larvae progressively destroy liver parenchyma through direct erosion and mechanical compression, leading to cirrhosis, portal hypertension and ultimately hepatic failure. With a 10-year mortality rate exceeding 90% in untreated cases, AE is termed “a helminthic infection with malignant characteristics” due to its metastatic potential via hematogenous or lymphatic dissemination to organs such as the lungs and brain, causing multiorgan failure [21–23]. This malignancy-like behavior underscores mechanistic parallels with tumor progression, suggesting that targeting host–parasite interactions may offer therapeutic breakthroughs [24].

Recent studies highlight the pivotal role of the CCL2–CCR2 axis in tumor invasion and metastasis, mediated by: (i) activation of PI3K-AKT signaling to promote extracellular matrix degradation and epithelial–mesenchymal transition (EMT) [24]; (ii) induction of M2-polarized tumor-associated macrophages (TAMs) that secrete immunosuppressive cytokines (e.g., interleukin [IL-10], tumor growth factor-beta [TGF-β]) and pro-angiogenic factors like VEGF, fostering immune-tolerant microenvironments [25]; and (iii) metabolic crosstalk between stromal cells (e.g. adipocytes, astrocytes) and tumor cells to fuel metastatic spread. Intriguingly, our study identified analogous activation of the CCL2–CCR2 axis in AE lesions, accompanied by PI3K-AKT pathway activation, M2 macrophage polarization and upregulated vascular markers CD31/VEGF in *E. multilocularis*-infected murine livers.

RS504393, a selective CCR2 antagonist, has demonstrated antitumor efficacy by reducing TAM infiltration and tumor burden in cancer models [26] while also ameliorating hepatic injury and fibrosis [27]. In the present study, RS504393 intervention significantly reduced parasitic load, attenuated fibrosis and improved liver function in infected mice. Mechanistically, RS504393 exerted multifaceted effects, as follows:

(i) Direct suppression of parasitic survival networks: the downregulation of VEGF/CD31 expression diminished lesion vascularization, disrupting nutrient supply and metastatic routes.
(ii) Alleviation of hepatic fibrosis: the inhibition of PI3K/AKT signaling suppressed α-SMA/TGF-β expression in HSCs, induced apoptosis (Bax↑/Bcl-2↓) and reduced serum fibrosis markers (HA, LN, PCIII, C-IV).The results of this study demonstrate that inhibition of the CCL2–CCR2/PI3K-AKT signaling axis significantly induced apoptosis of activated HSCs, representing a core mechanism mitigating the progression of fibrosis. However, it is well-established that the reversal of hepatic fibrosis is exceedingly challenging and its subsequent regression is an active process. This requires not only the cessation of extracellular matrix production but also its enzymatic clearance [28, 29]. The results of the MMP1 IHC assay presented in Additional file 1: Figure S1 confirmed that following RS504393 intervention, the MMP1 IHC-positive area was significantly reduced (following RS504393 intervention, the significant reduction in MMP1-positive areas observed through IHC reflects a attenuation of the disease's driving forces—specifically, the effective suppression of macrophage infiltration and hepatic stellate cell activation via blockade of the CCL2–CCR2 axis, leading to attenuated fibrosis and a consequent reduction in degradable collagen). This suggests that the attenuation of hepatic fibrosis following blockade of the CCL2–CCR2 signaling pathway partly relies on the coordinated action of MMPs to degrade excessive collagenous matrix. Furthermore, it has been documented that a therapeutic strategy combining inhibition of both the CCL2–CCR2 pathway and MMP1 can suppress intrahepatic monocyte recruitment, enhance collagen degradation and concurrently ameliorate liver inflammation and fibrosis [30]. Building on existing research, it has also been reported that macrophages from CCL2–CCR2-deficient mice exhibit weaker expression of MMP-2 and MMP-9, accompanied by reduced hepatic fibrosis in mouse models [31]. Collectively, the aforementioned literature and our supplementary experimental data indicate that blocking CCL2–CCR2 alleviates hepatic fibrosis in *E. multilocularis*-infected mice likely through a dual mechanism: synergistically promoting HSC apoptosis and facilitating MMP-mediated degradation of the extracellular matrix;
(iii) Immune microenvironment remodeling: reduced Arg-1 (an M2 marker) and IL-10 levels reversed immunosuppression. These findings align with RS504393's antitumor mechanisms [32, 33], but uniquely highlight its potential in parasitic diseases by targeting host pathways rather than direct parasite lethality—a strategy that may circumvent drug resistance.

Notably, *E. multilocularis* directly secretes VEGF-like factors to promote angiogenesis [32, 33]. Our study revealed that RS504393 not only inhibits host-derived VEGF but may also interfere with parasite-driven angiogenic networks, offering a dual therapeutic approach. Furthermore, PI3K-AKT pathway inhibition concurrently blocks HSC activation, M2 polarization and angiogenesis, underscoring its central role in AE pathogenesis.

This study systematically elucidates the multimodal therapeutic mechanisms of RS504393 via CCL2–CCR2/PI3K-AKT axis blockade. Bioinformatics and molecular docking confirmed high-affinity CCL2–CCR2 interactions linked to PI3K-AKT activation, mirroring chemokine signaling roles in fibrosis and tumor microenvironments but first associating it with parasite-driven pathology. Histopathological evidence revealed co-activation of CCL2–CCR2 and PI3K-AKT in clinical and murine AE samples, suggesting this axis facilitates immune evasion and fibrotic niche formation. By integrating these insights, we propose RS504393 as a novel host-directed therapy for AE, providing a framework for non-surgical management of advanced cases. Most importantly, the rescue experiment demonstrated that exogenous activation of the PI3K-AKT pathway completely reversed the anti-fibrotic and pro-apoptotic effects of RS504393. This finding provides direct causal evidence supporting the mechanism that RS504393 acts through inhibiting the PI3K-AKT pathway, thereby strengthening the rigor of our conclusion (Additional file 3: Figure S3). Future studies should address dynamic CCL2–CCR2 regulation across parasitic life stages, long-term immune impacts and crosstalk with other profibrotic pathways (e.g. TGF-β/Smad). Scanning electron microscopy (SEM) studies have shown that treatment with benzimidazole derivatives causes significant fissures and defects in the laminated layer of *E. multilocularis* PSCs, reducing the germinal layer thickness by > 70% and disrupting morphological integrity. In *E. multilocularis*-infected mice, benzimidazole derivatives reduce the number of microcysts, most of which become hardened, markedly calcified and coagulated; cells within the cyst wall are scattered, with some undergoing necrosis and vacuolization. These drugs inhibit β-tubulin polymerization, disrupting microtubule function in* Echinococcus*, impairing secretory granule transport and other organelle movements and inhibiting glucose uptake by the parasite, leading to glycogen depletion [34]. Similarly, SEM studies have shown that treatment of *E. multilocularis* PSCs with benzimidazole derivatives results in rostellar hook contraction, loss of the distinct laminated structure of the outer layer and a significant reduction in the number of PSCs and daughter cysts within the germinal layer. In mouse models of *E. multilocularis* infection, benzimidazole derivative intervention reduces parasitic load [35]. Benzimidazole derivatives are relatively insoluble in water and most organic solvents, making them difficult to absorb from the gastrointestinal tract unless administered with a high-fat meal. The oral absorption rates of albendazole and mebendazole in the human intestine are only about 1–5%, resulting in a cure rate for echinococcosis of approximately 30%. Furthermore, 20–40% of patients respond poorly to treatment and experience significant side effects. The limited solubility and dissolution rate (< 5%) of these drugs lead to poor bioavailability and a high disease recurrence rate post-treatment. Moreover, these drugs primarily exhibit parasitic effects rather than complete parasiticidal activity, often necessitating long-term therapy, which can cause significant toxicities, such as hepatotoxicity and bone marrow suppression. Additionally, as these drugs cannot fully eradicate the parasite, treatment cessation often leads to high recurrence rates [36–39]. Furthermore, studies have shown that mice genetically deficient in CCL2 or its receptor CCR2 exhibit diminished hepatic damage compared with wild-type mice following ischemia/reperfusion (I/R) injury, by controlling intrahepatic inflammatory Ly6Chi monocyte accumulation; moreover, the specific CCR2 inhibitor RS504393 alleviated hepatic I/R injury. These results suggest that the CCL2–CCR2 axis plays an important role in monocyte infiltration and may represent a novel target for treating liver I/R injury [40]. In our current study, RS504393 effectively promoted fibrosis attenuation. Furthermore, supplementary in vitro experiments demonstrated that RS504393 exerts a direct effect on the parasite itself, manifested as a time-dependent reduction in protoscolex viability and the induction of oxidative stress (Additional file 4–5: Figure S4–S5). Therefore, a combination therapy strategy holds promise for significantly enhancing the intralesional penetration concentration of benzimidazole drugs by degrading fibrous septa, thereby leading to more effective parasite killing. Such a combination therapy strategy has a potential multi-target synergistic effect: benzimidazoles target the parasite itself, while the CCR2 inhibitor addresses host-specific pathogenic mechanisms (such as angiogenesis, HSC activation and immunosuppression). This dual-pronged strategy—simultaneously attacking the parasite and altering its permissive host microenvironment—may provide a novel pathway for achieving more complete parasitological clearance and preventing recurrence. Combinatorial strategies with albendazole or PI3K-AKT-mTOR inhibitors may further optimize therapeutic outcomes.

## Conclusions

This study elucidates the therapeutic efficacy of the CCR2 antagonist RS504393 against AE through multimodal mechanisms. Integrated bioinformatics and molecular docking revealed high-affinity CCL2–CCR2 interaction, with transcriptomic profiling implicating chemokine signaling and PI3K-AKT pathway activation in AE progression. Histopathological analyses of human and murine AE lesions demonstrated hyperactivation of the CCR2–CCL2 axis, correlating with hepatic fibrosis, M2 macrophage polarization and PI3K-AKT upregulation. RS504393 intervention significantly reduced parasitic burden and fibrosis in *E. multilocularis*-infected mice by suppressing CCR2–CCL2/PI3K-AKT signaling. Mechanistically, RS504393 attenuated angiogenesis via downregulation of CD31/VEGF in endothelial progenitor cells, induced hepatic stellate cell apoptosis and reversed M2 macrophage polarization. Multicellular co-culture models confirmed RS504393's coordinated effects: inhibition of PI3K-AKT-driven macrophage M2 polarization, promotion of stellate cell apoptosis and impairment of pro-angiogenic activity. These findings establish CCL2–CCR2 blockade as a dual-pronged strategy to disrupt parasitic metabolic support and fibrotic remodeling, providing a foundation for targeted therapies against AE-associated liver pathology.

## Supplementary Information

Additional file 1: Figure S1. Immunohistochemical analysis and quantification of MMP1 and MMP8 in DLT and CLT liver tissues from patients with AE.A Representative IHC images of MMP1 expression in DLT and CLT tissues (*n* = 10 per group). B Representative IHC images of MMP8 expression in DLT and CLT tissues (*n* = 10 per group). C Quantitative analysis of IHC staining for MMP1 and MMP8. Data are expressed as the mean ± SD. *P < 0.05, **P < 0.01, ***P < 0.001 by two-tailed paired t-test.

Additional file 2: Figure S2. Expression of p-PI3K and p-AKT proteins in AE patient tissues, mouse liver tissues, and various cell lines. A, B Western blot analysis (left) and quantitative analysis (right) of the p-PI3K/PI3K and p-AKT/AKT ratios in DLT and CLT liver tissues from AE patients (*n* = 10 per group). C, D Western blot analysis (left) and quantitative analysis (right) of the p-PI3K/PI3K and p-AKT/AKT ratios in liver tissues from mice in each group (*n* = 5 per group). E, F Western blot analysis (left) and quantitative analysis (right) of the p-PI3K/PI3K and p-AKT/AKT ratios in RAW264.7 cells from each group (*n* = 3 per group). G, H Western blot analysis (left) and quantitative analysis (right) of the p-PI3K/PI3K and p-AKT/AKT ratios in JS1 cells from each group (*n* = 3 per group). Data are presented as the mean ± SEM. Statistical comparisons among groups were performed using one-way ANOVA with appropriate post hoc tests for multiple comparisons. *P < 0.05, **P < 0.01, ***P < 0.001.

Additional file 3: Figure S3. Activation of the PI3K-AKT pathway by 740 Y-P reverses the anti-fibrotic and pro-apoptotic effects of RS504393. A, B Western blot analysis (left) and quantitative analysis (right) of the p-PI3K/PI3K and p-AKT/AKT ratios in RAW264.7 cells from each group (*n* = 3 per group). C, D Western blot analysis (left) and quantitative analysis (right) of the Arg-1 and INOS/GAPDH ratios in RAW264.7 cells and the α-SMA, CollagenI, Bcl2 and Cleaved-caspased 3/GAPDH ratios in JS1 cells from each group (*n* = 3 per group). Data are presented as the mean ± SEM. Statistical comparisons among groups were performed using one-way ANOVA with appropriate post hoc tests for multiple comparisons. *P < 0.05, **P < 0.01, ***P < 0.001.

Additional file 4: Figure S4. In vitro effects of the RS504393 inhibitor on *Echinococcus multilocularis* (Viability of *E. multilocularis* protoscoleces co-cultured with RS504393 or Albendazole for 7 days, assessed by trypan blue staining) (*n* = 3 per group).

Additional file 5: Figure S5. Results of ROS fluorescence assay in *Echinococcus multilocularis* protoscoleces after 7-day culture with RS504393 or Albendazole (*n* = 3 per group).

Additional file 5: Figure S5. Results of ROS fluorescence assay in *Echinococcus multilocularis* protoscoleces after 7-day culture with RS504393 or Albendazole (*n* = 3 per group).

## Data Availability

All relevant data generated in this study are present within the manuscript and Supplemental Information.
