# Correction: Blockade of the CCL2–CCR2 axis attenuates fibrosis and parasite load in vesicular echinococcosis by inhibiting the PI3K-AKT pathway that regulates angiogenesis and hepatic stellate cell apoptosis

**DOI:** 10.1186/s13071-026-07512-z

**Published:** 2026-08-03

**Authors:** Bin Fan, Yuyu Ma, Menggen Meng, Jiahui Chen, Xinwei Qi, Yuqin Sun, Xuan Zhou, Haonan Wang, Xiumin Ma, Liang Wang

**Affiliations:** 1 Basic Medical College of Xinjiang Medical University, Ürümqi, Xinjiang PR China; 2 State Key Laboratory of Pathogenesis, Prevention and Treatment of High Incidence Diseases in Central Asia, Clinical Laboratory Center, Tumor Hospital Affiliated to Xinjiang Medical University, Ürümqi, 830011 Xinjiang PR China; 3 Medical Testing Center, The Fifth Affiliated Hospital of Xinjiang Medical University, Ürümqi, 830011 Xinjiang PR China; 4 Medical Testing Center, The First Affiliated Hospital of Xinjiang Medical University, Ürümqi, Xinjiang PR China

**Correction: Parasites & Vectors (2026) 19:239** 10.1186/s13071-026-07306-3

Following publication of the article [1], it was brought to the attention of the journal that Figure 2 (specifically, panel 2F of the figure) was incorrect. The figure has since been corrected in the article. Thank you for reading this erratum and apologies for any inconvenience caused.

## References

[1] Fan B, Ma Y, Meng M, Chen J, Qi X, Sun Y, et al. Blockade of the CCL2–CCR2 axis attenuates fibrosis and parasite load in vesicular echinococcosis by inhibiting the PI3K-AKT pathway that regulates angiogenesis and hepatic stellate cell apoptosis. Parasites Vectors. 2026;19:239. 10.1186/s13071-026-07306-3. 42057202 10.1186/s13071-026-07306-3 PMC13227867

