# Supplementary figures and images for: Blockade of the CCL2–CCR2 axis attenuates fibrosis and parasite load in vesicular echinococcosis by inhibiting the PI3K-AKT pathway that regulates angiogenesis and hepatic stellate cell apoptosis
Source: Parasit Vectors. 2026 Apr 29;19:239. doi: 10.1186/s13071-026-07306-3 (PMC13227867; doi:10.1186/s13071-026-07306-3)

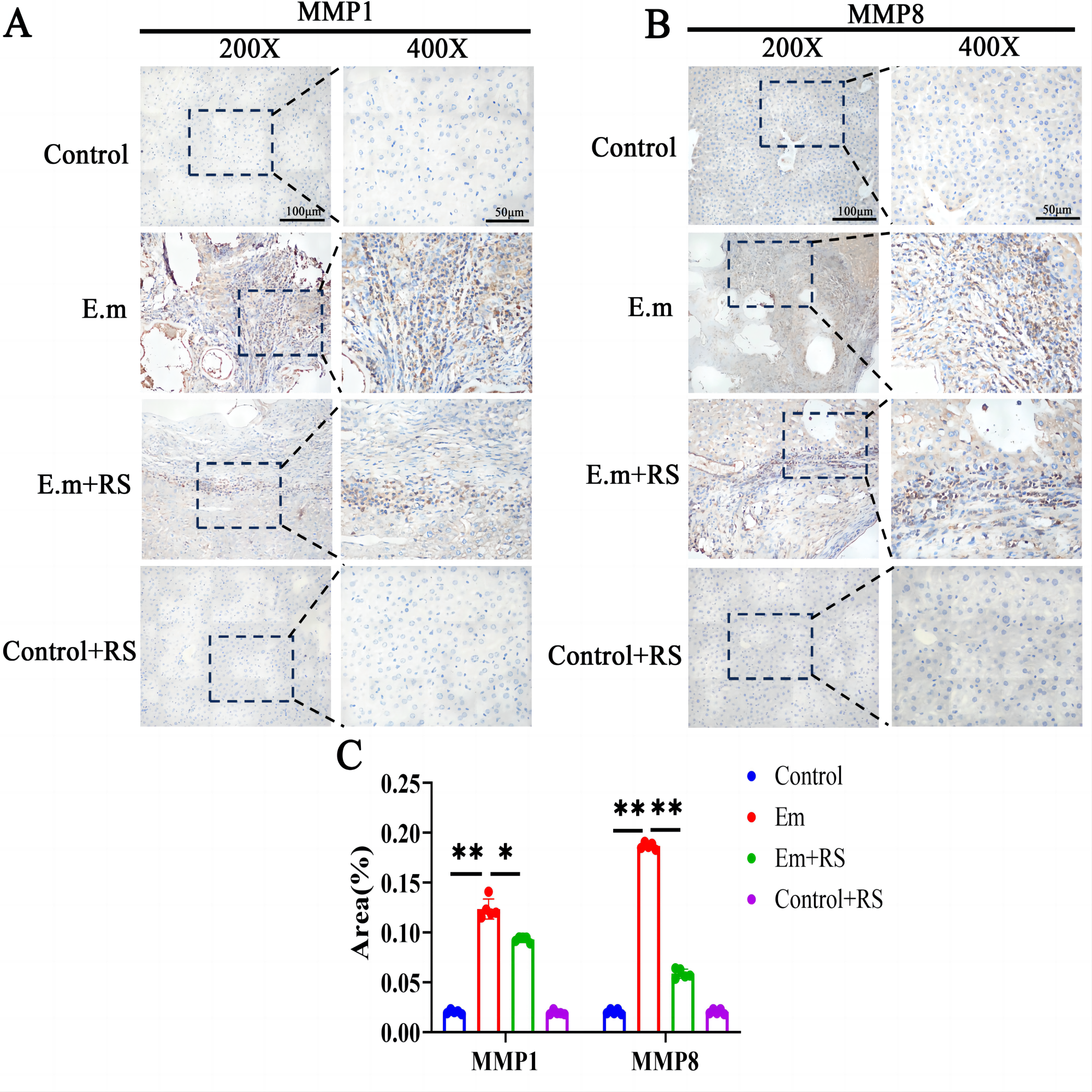

Supplement: Supplementary file 1 — Additional file 1: Figure S1. Immunohistochemical analysis and quantification of MMP1 and MMP8 in DLT and CLT liver tissues from patients with AE.A Representative IHC images of MMP1 expression in DLT and CLT tissues (n = 10 per group). B Representative IHC images of MMP8 expression in DLT and CLT tissues (n = 10 per group). C Quantitative analysis of IHC staining for MMP1 and MMP8. Data are expressed as the mean ± SD. *P < 0.05, **P < 0.01, ***P < 0.001 by two-tailed paired t-test. [file 13071_2026_7306_MOESM1_ESM.tif]

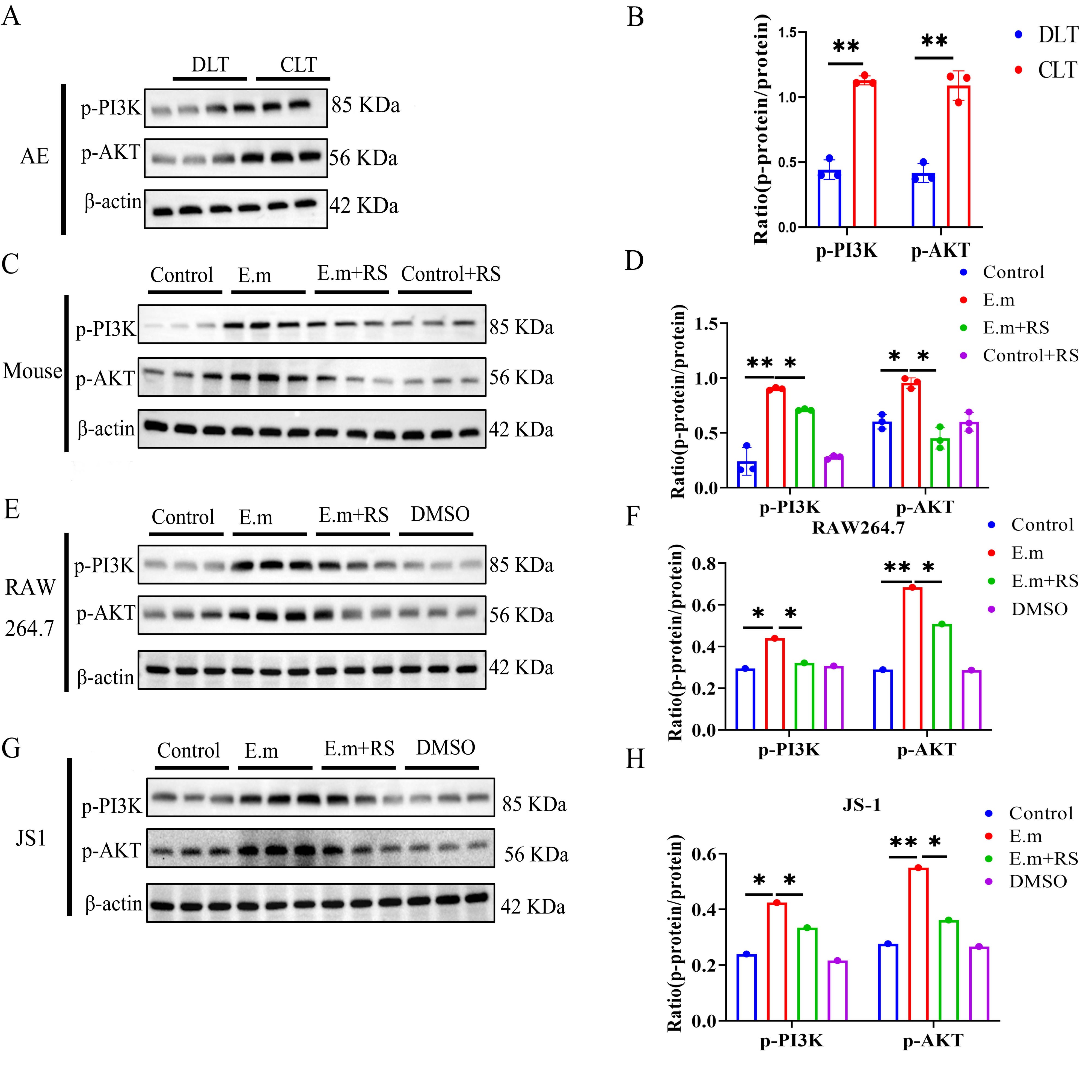

Supplement: Supplementary file 2 — Additional file 2: Figure S2. Expression of p-PI3K and p-AKT proteins in AE patient tissues, mouse liver tissues, and various cell lines. A, B Western blot analysis (left) and quantitative analysis (right) of the p-PI3K/PI3K and p-AKT/AKT ratios in DLT and CLT liver tissues from AE patients (n = 10 per group). C, D Western blot analysis (left) and quantitative analysis (right) of the p-PI3K/PI3K and p-AKT/AKT ratios in liver tissues from mice in each group (n = 5 per group). E, F Western blot analysis (left) and quantitative analysis (right) of the p-PI3K/PI3K and p-AKT/AKT ratios in RAW264.7 cells from each group (n = 3 per group). G, H Western blot analysis (left) and quantitative analysis (right) of the p-PI3K/PI3K and p-AKT/AKT ratios in JS1 cells from each group (n = 3 per group). Data are presented as the mean ± SEM. Statistical comparisons among groups were performed using one-way ANOVA with appropriate post hoc tests for multiple comparisons. *P < 0.05, **P < 0.01, ***P < 0.001. [file 13071_2026_7306_MOESM2_ESM.tif]

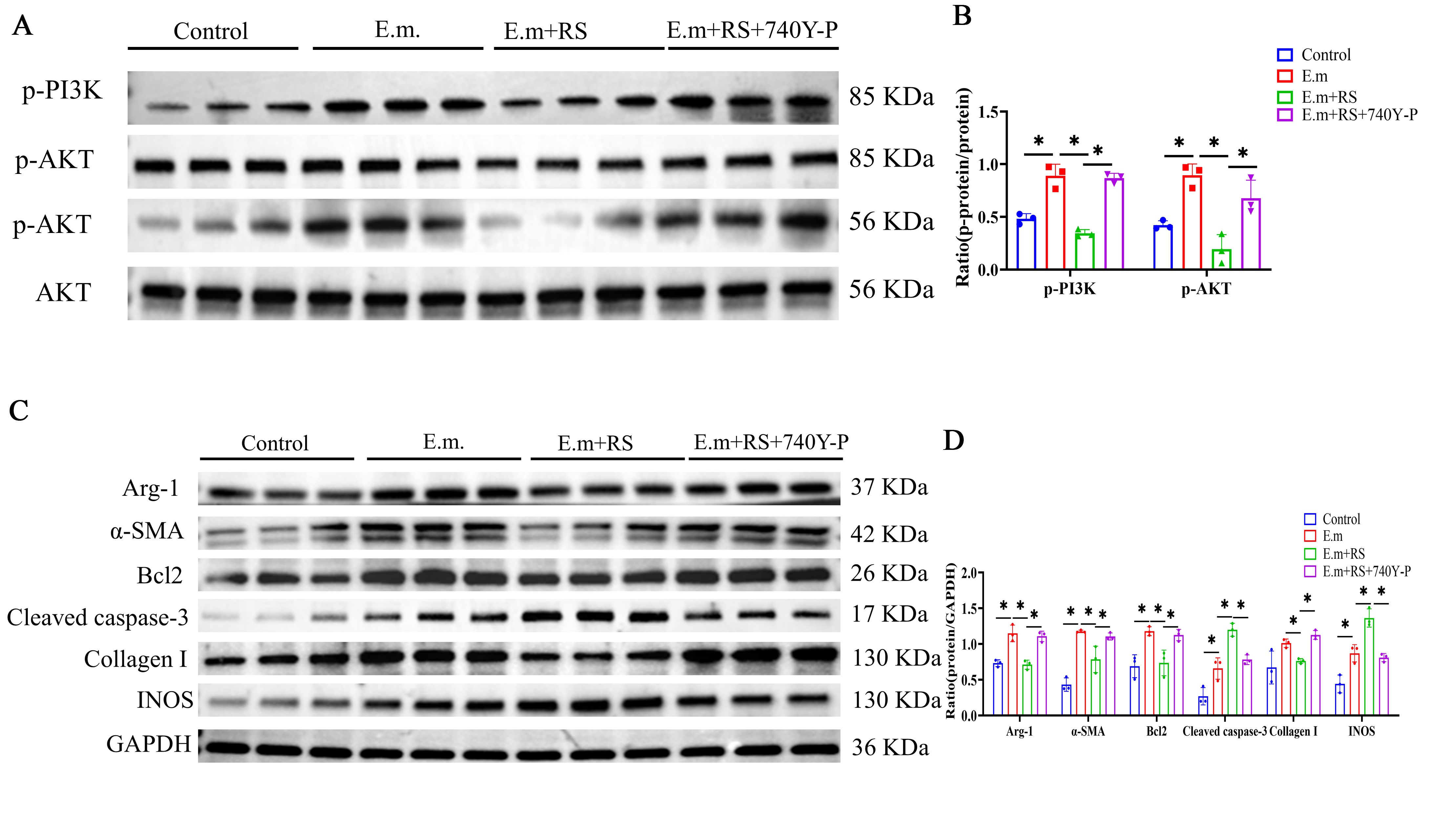

Supplement: Supplementary file 3 — Additional file 3: Figure S3. Activation of the PI3K-AKT pathway by 740 Y-P reverses the anti-fibrotic and pro-apoptotic effects of RS504393. A, B Western blot analysis (left) and quantitative analysis (right) of the p-PI3K/PI3K and p-AKT/AKT ratios in RAW264.7 cells from each group (n = 3 per group). C, D Western blot analysis (left) and quantitative analysis (right) of the Arg-1 and INOS/GAPDH ratios in RAW264.7 cells and the α-SMA, CollagenI, Bcl2 and Cleaved-caspased 3/GAPDH ratios in JS1 cells from each group (n = 3 per group). Data are presented as the mean ± SEM. Statistical comparisons among groups were performed using one-way ANOVA with appropriate post hoc tests for multiple comparisons. *P < 0.05, **P < 0.01, ***P < 0.001. [file 13071_2026_7306_MOESM3_ESM.tif]

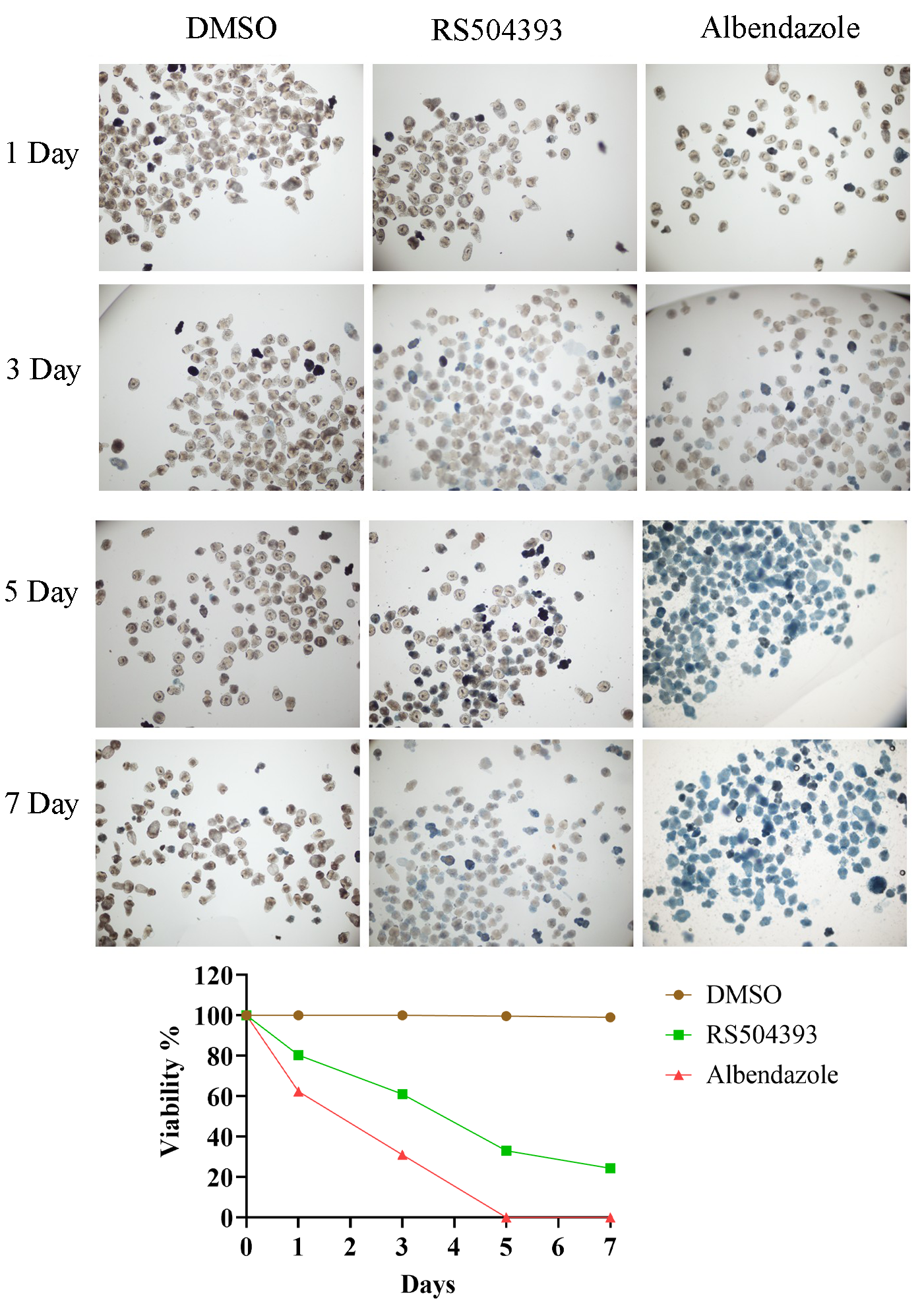

Supplement: Supplementary file 4 — Additional file 4: Figure S4. In vitro effects of the RS504393 inhibitor on Echinococcus multilocularis (Viability of E. multilocularis protoscoleces co-cultured with RS504393 or Albendazole for 7 days, assessed by trypan blue staining) (n = 3 per group). [file 13071_2026_7306_MOESM4_ESM.tif]

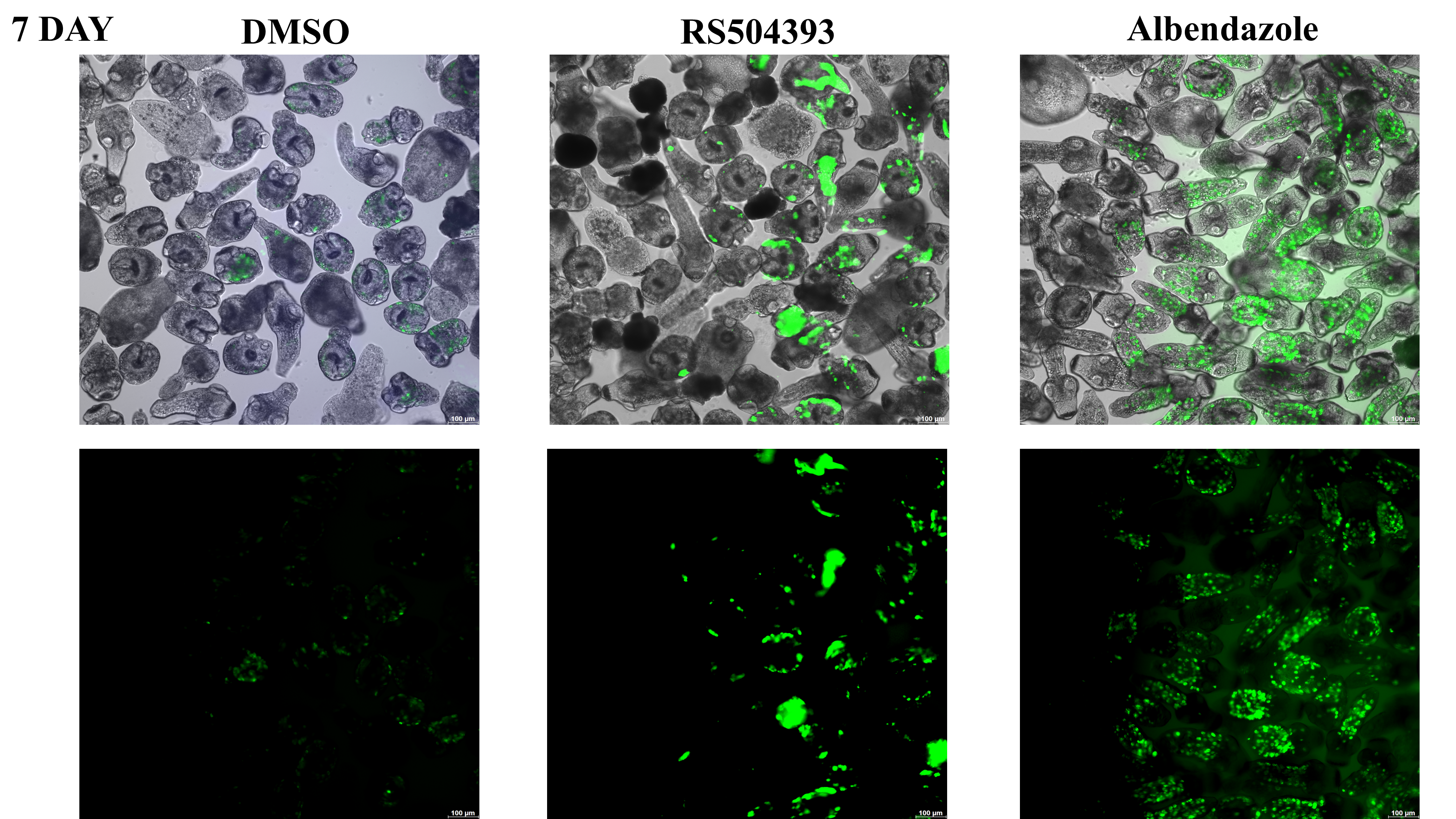

Supplement: Supplementary file 5 — Additional file 5: Figure S5. Results of ROS fluorescence assay in Echinococcus multilocularis protoscoleces after 7-day culture with RS504393 or Albendazole (n = 3 per group). [file 13071_2026_7306_MOESM5_ESM.tif]
