# Supplementary material for: Blockade of the CCL2–CCR2 axis attenuates fibrosis and parasite load in vesicular echinococcosis by inhibiting the PI3K-AKT pathway that regulates angiogenesis and hepatic stellate cell apoptosis
Source: Parasit Vectors. 2026 Apr 29;19:239. doi: 10.1186/s13071-026-07306-3 (PMC13227867; doi:10.1186/s13071-026-07306-3)
Supplement: Supplementary file 6 — Additional file 5: Figure S5. Results of ROS fluorescence assay in Echinococcus multilocularis protoscoleces after 7-day culture with RS504393 or Albendazole (n = 3 per group). [file 13071_2026_7306_MOESM6_ESM.docx]

**Table S1**

**Supplementary description of experimental reagents**

| **Antibody** | **Source** | **Catalog Number** |
| --- | --- | --- |
| RS504393 | MCE | Cat# HY-15418 |
| ELISA kits | BOSTER BIOLOGICAL TECHNOLOGY Co., Ltd. |  |
| BM-EPCs cell | Wuhan Presla Biotechnology Co., Ltd. | Cat# CP-M140 |
| JS1 cell | Wuhan Presla Biotechnology Co., Ltd. | Cat# CP-M041 |
| RAW 264.7 | Wuhan Presla Biotechnology Co., Ltd. | Cat# CL-0190 |
| CCK-8 reagent | proteintech | PF00004 |
| the FITC Annexin V Apoptosis Detection Kit I | BD Biosciences | Cat# 556547 |
| hematoxylin-eosin (HE) kits | Solarbio | G1120 |
| Sirius red staining kit | Shanghai Maokang Biotechnology | MM1036 |
| the PrimeScript™ RT Reagent Kit with gDNA Eraser | Takara Bio | RR047A |
| TB Green® Premix Ex Taq™ II | Takara Bio | RR820A |
| BCA assay kits | Solarbio | Cat# PC0020 |

**Table S2**

**Supporting Information Antibodies for ELISA**

**（****BOSTER BIOLOGICAL TECHNOLOGY Co., Ltd.）**

| **Antibody** | **Source** | **Catalog Number** |
| --- | --- | --- |
| IL-2 | BOSTER | MEK2007 |
| IL-4 | BOSTER | MEK2007 |
| IL-10 | BOSTER | MEK2007 |
| IFN-γ | BOSTER | MEK2007 |
| TGF-β1 | BOSTER | MEK2007 |

**Table S3**

**Information Antibodies for immunohistochemistry (IHC)**

| **Antibody** | **Source** | **Catalog Number** | **Concentration** |
| --- | --- | --- | --- |
| α-SMA | Bioss | Bsm-33188M | 1:200 |
| CollagenⅠ | Affinity | AF7001 | 1:100 |
| TGF-β1 | Bioss | bs-0086R | 1:200 |
| Arg-1 | proteintech | 16001-1-AP | 1:5000 |
| CCR2 | Wanleibio | WL03663 | 1:200 |
| CCL2 | Wanleibio | WLH3384 | 1:200 |
| PI3K | Affinity | AF6241 | 1:100 |
| AKT | Abcam | ab179463 | 1:250 |
| CD31 | proteintech | 28083-1-AP | 1:5000 |
| VEGF | proteintech | 19003-1-AP | 1:1000 |
| Goat anti-rabbit/mouse HRP-labeled polymer | proteintech | PK10006 |  |

**Table S4**

**Supporting Information Primer sequences for qRT-PCR**

**Primer sequence of human**

| **Gene（human）** | **（5’→3’）** |
| --- | --- |
| hα-SMA | Forward: CCCAGACATCAGGGAGTAATGG |
|  | Reverse: TCTATCGGATACTTCAGCGTCA |
| hTGF-β | Forward: GGCCAGATCCTGTCCAAGC |
|  | Reverse: GTGGGTTTCCACCATTAGCAC |
| hArg-1 | Forward: GTGGAAACTTGCATGGACAAC |
|  | Reverse: AATCCTGGCACATCGGGAATC |
| hCCL2 | Forward: CAGCCAGATGCAATCAATGCC |
|  | Reverse: TGGAATCCTGAACCCACTTCT |
| hCCR2 | Forward: CCACATCTCGTTCTCGGTTTATC |
|  | Reverse: CAGGGAGCACCGTAATCATAATC |
| hPI3K | Forward: TATTTGGACTTTGCGACAAGACT |
|  | Reverse: TCGAACGTACTGGTCTGGATAG |
| hAKT | Forward: AGCGACGTGGCTATTGTGAAG |
|  | Reverse: GCCATCATTCTTGAGGAGGAAGT |
| hGAPDH | Forward: GCACCGTCAAGGCTGAGAAC |
|  | Reverse: TGGTGAAGACGCCAGTGGA |

**Primer sequence of mouse**

| **Gene（mouse）** | **（5’→3’）** |
| --- | --- |
| mα-SMA | Forward: TTCGTGACTACTGCCGAGC |
|  | Reverse: GTCAGGCAGTTCGTAGCTCT |
| mTGF-β1 | Forward: GTGTGGAGCAACATGTGGAACTCTA |
|  | Reverse: TTGGTTCAGCCACTGCCGTA |
| mArg-1 | Forward: CTCCAAGCCAAAGTCCTTAGAG |
|  | Reverse: GGAGCTGTCATTAGGGACATCA |
| mCCL2 | Forward: TTAAAAACCTGGATCGGAACCAA |
|  | Reverse: GCATTAGCTTCAGATTTACGGGT |
| mCCR2 | Forward: ATCCACGGCATACTATCAACATC |
|  | Reverse: CTGCCTGCGACAGATGAGTG |
| mPI3K | Forward: TCCGATTACCAAGTGCTCTTTC |
|  | Reverse: GCAGCAAAGGCTTCTGGGATAA |
| mAKT | Forward: GTCATCGAACGCACCTTCCAT |
|  | Reverse: AGCTTCAGGTACTCAAACTCGT |
| mCD31 | Forward: ACGCTGGTGCTCTATGCAAG |
|  | Reverse: TCAGTTGCTGCCCATTCATCA |
| mVEGF | Forward: CTGCCGTCCGATTGAGACC |
|  | Reverse: CCCCTCCTTGTACCACTGTC |
| mBcl2 | Forward: GTCGCTACCGTCGTGACTTC |
|  | Reverse: CAGACATGCACCTACCCAGC |
| mBax | Forward: TGAAGACAGGGGCCTTTTTG |
|  | Reverse: AATTCGCCGGAGACACTCG |
| mCaspase | Forward: GCGCCGGTTGAAGATGACA |
|  | Reverse: TGCAGAGCTAAGGAGGACACT |
| mGAPDH | Forward: AGGTCGGTGTGAACGGATTTG |
|  | Reverse: GGGGTCGTTGATGGCAACA |

**Table S5**

**Supporting Information Antibodies for Western blot (WB)**

| **Antibody** | **Source** | **Catalog Number** | **Concentration** |
| --- | --- | --- | --- |
| α-SMA | Bioss | Bsm-33188M | 1:1000 |
| TGF-β1 | Bioss | bs-0086R | 1:1000 |
| Arg-1 | proteintech | 16001-1-AP | 1:5000 |
| CCR2 | Wanleibio | WL03663 | 1:500 |
| CCL2 | Wanleibio | WL02966 | 1:500 |
| p-PI3K | Abcam | ab191606 | 1:1000 |
| PI3K | Abcam | ab191606 | 1:1000 |
| AKT | Abcam | ab179463 | 1:5000 |
| p-AKT | Abcam | ab192623 | 1:1000 |
| CD31 | proteintech | 28083-1-AP | 1:5000 |
| VEGF | Abcam | 19003-1-AP | 1:10000 |
| BCL2 | proteintech | 26593-1-AP | 1:1500 |
| Bax | Abcam | Ab32503 | 1:5000 |
| cleaved-caspase3 | proteintech | 25128-1-AP | 1:1000 |
| Multi-rAb® HRP-Goat Anti-Mouse Recombinant Secondary Antibody (H+L) | proteintech | RGAM001 | 1:8000 |
| MMP1 | proteintech | 10371-2-AP | 1:400 |
| MMP8 | proteintech | 17874-1-AP | 1:100 |
| Goat Anti-Rabbit IgG H&L, HRP conjugated（） | BIOSS | bs-0295G-HRP | 1:17000 |
